# Design of a Fluorescence Polarization Probe for Enterovirus 2C Proteins

**DOI:** 10.1021/acs.jmedchem.5c01219

**Published:** 2025-06-21

**Authors:** Kan Li, Hiwot A Demssie, Jun Wang

**Affiliations:** Department of Medicinal Chemistry, Ernest Mario School of Pharmacy, 242612 Rutgers, The State University of New Jersey, Piscataway, New Jersey 08854, United States

## Abstract

Enteroviruses (EVs), such as EV-D68, EV-A71, and CVB3, cause significant human disease; yet, no antivirals are currently approved. The highly conserved 2C protein, an essential AAA+ ATPase and helicase, is a prime antiviral target; however, it lacks suitable assays for inhibitor screening. Here, we report a fluorescence polarization (FP) assay using a rationally designed probe, **Jun14157**, which binds a conserved allosteric site in 2C with high affinity. This assay enables the quantitative assessment of binding to diverse 2C inhibitors with high signal-to-background ratios, DMSO tolerance, and a strong correlation between FP *K*_i_ and cellular EC_50_. Using this platform, we validated hits from virtual screening and identified two novel inhibitors, **Jun15716** and **Jun15799**. This FP assay offers a robust and scalable tool for the mechanistic characterization and high-throughput screening of 2C-targeting antivirals.

## Graphical abstract

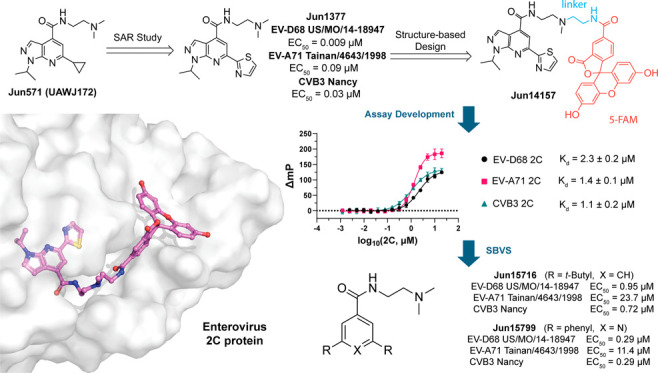

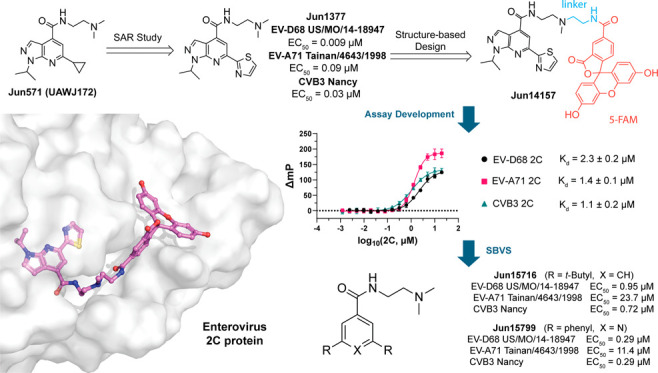

## Introduction

Enteroviruses (EVs), members of the *Picornaviridae* family, are small, nonenveloped, positive-sense single-stranded RNA viruses.^1,2^ Infections caused by different EV species result in a range of clinical manifestations, from mild illnesses to severe conditions. Enterovirus D68 (EV-D68) was historically associated with mild flu-like symptoms (e.g., the Fermon strain), but more recent outbreaks in North America involved contemporary strains with enhanced virulence. These new EV-D68 strains led to severe respiratory illnesses and neurological complications, including acute flaccid myelitis (AFM), meningitis, and encephalitis.^3–7^ Enterovirus A71 (EV-A71) is a primary causative agent of hand, foot, and mouth diseases (HFMD), predominantly affecting children. It is mainly transmitted via the fecal-oral route.^8^ Several large EV-A71 outbreaks in the Asia-Pacific region have resulted in thousands of deaths.^9^ Coxsackievirus B3 (CVB3) is a major pathogen for viral myocarditis^10,11^ and has also been linked to type I diabetes mellitus and idiopathic chronic pancreatitis.^12^ Currently, there is no FDA-approved antiviral treatment against EVs. In China, three inactivated EV-A71 vaccines have been approved; however, their protective efficacy is limited due to the lack of broad-spectrum protection.^13–15^

The EV genome is approximately 7.4 kb long and encodes a single open reading frame (ORF), flanked by untranslated regions (UTRs) at 5′ and 3′ ends. The ORF consists of three main genomic regions, namely, P1 encoding viral capsid proteins VP1–VP4, P2 encoding nonstructural proteins 2A, 2B, and 2C, and P3 encoding nonstructural proteins 3A, 3B, 3C, and 3D (Figure 1A).^16–18^ Among these, 2C is a high-profile antiviral drug target due to its essential role in viral replication and minimal sequence similarity to host proteins.^19–21^ The 2C protein soluble domain consists of 330 residues and contains a cysteine-rich zinc finger, a C-terminal helical domain, and an adenosine triphosphatase (ATPase) domain (Figure 1A).^22^ An allosteric drug binding site is located adjacent to the ATP-binding pocket (Figure 1B).^23^ 2C possesses ATPase and helicase activities. It is involved in multiple processes, including viral genome replication, the encapsulation of new viral particles, host cell membrane rearrangement, and viral uncoating.^24^ Given its high structural conservation and sequence homology among EVs (Figure S1), 2C represents a high-profile target for broad-spectrum antiviral development.^21^

**Figure 1:**
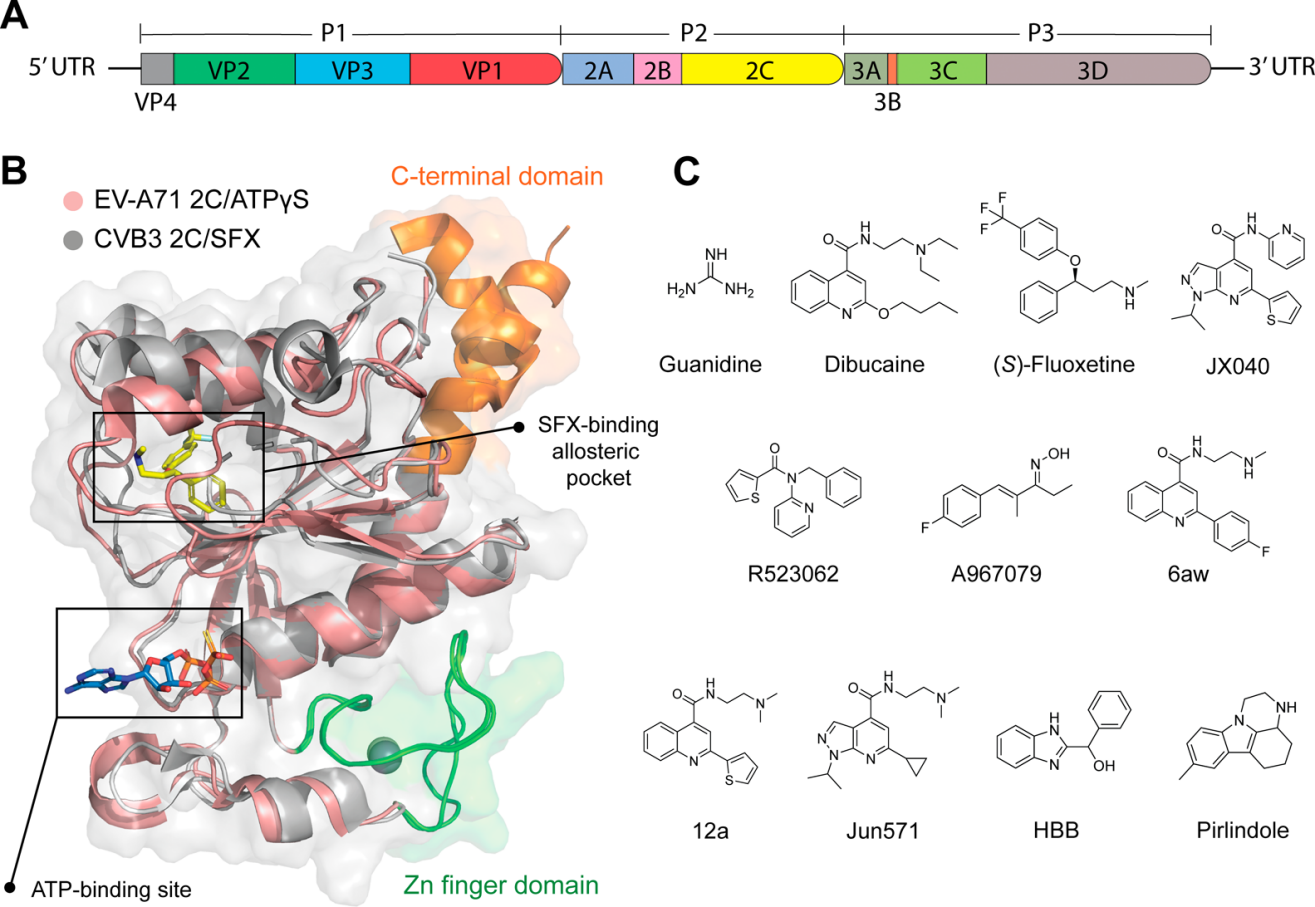
Genome structure of enterovirus, structure of 2C, and reported 2C inhibitors. (A) The EV genome contains ORF encoding structural proteins VP1, VP2, VP3, and VP4 and nonstructural proteins 2A, 2B, and 2C and 3A, 3B, 3C, and 3D. (B) Alignment of the crystal structure of CVB3 2C in complex with SFX (PDB ID 6S3A, colored in gray) and EV-A71 2C in complex with ATPγS (PDB ID 5GRB, colored in wheat). The C-terminal part of 2C is highlighted in orange, the zinc finger domain is highlighted in green, ATPγS is shown in blue sticks, and SFX is shown in yellow sticks. (C) Chemical structures of reported enterovirus 2C inhibitors.

Various 2C inhibitors have been reported with antiviral activity, including guanidine, dibucaine, (*S*)-fluoxetine (SFX), JX040, R523062, A967079, 6aw, 12a, **Jun571**, HBB [2-(α-hydroxybenzyl)-benzimidazole], and pirlindole (Figure 1C).^25–32^ All these 2C inhibitors were identified from phenotypic antiviral screening followed by target deconvolution through resistance selection.^18,22^ SFX binds to the allosteric pocket as revealed in the crystal structure of CVB3 2C (Figure 1B).^23,33^ The binding sites of the remaining 2C inhibitors have not yet been confirmed through structural biology. The 2C ATPase assay was previously used to evaluate 2C inhibitors using an engineered hexΔ116 CVB3 2C protein, which was constructed to form a stable hexameric structure.^23,34,35^ However, we failed to validate the ATPase inhibition of guanidine, dibucaine, SFX, JX040, R523062, A967079, 6aw, 12a, and **Jun571** with our truncated EV-D68 2C construction (residues 40–330) (Figure S2).^31^ It is reported that the absence of the N-terminal amphipathic helix (residues 1–38) can disrupt 2C homo-oligomer formation, which is essential for ATPase activity.^21,36^ The lack of inhibition observed for these 2C inhibitors in our study may be attributed to the intrinsically low ATPase activity of the truncated 2C proteins used. Therefore, further studies are necessary to optimize the ATPase assay for testing 2C inhibitors. For the binding assay of 2C, differential scanning fluorimetry (DSF) or thermal shift assay (TSA) was also used as an alternative biophysical technique to assess inhibitor binding by measuring melting temperature shifts (Δ*T*_m_) with and without the inhibitor.^27^ However, TSA lacks the sensitivity to quantify the binding affinity. Thus, a more direct, high-sensitivity, and broadly applicable binding assay is needed to facilitate the characterization of 2C allosteric inhibitors across various EVs.

Herein, we designed a novel 2C fluorescence probe, **Jun14157**, based on a potent pyrazolopyridine-based 2C allosteric inhibitor, **Jun1377**, which showed broad-spectrum antiviral activities against EV-D68, EV-A71, and CVB3. Using this FP probe, we developed a fluorescence polarization (FP) assay to quantify the binding affinities of allosteric inhibitors to EV-D68 2C, EV-A71 2C, and CVB3 2C. The assay was validated with several reported 2C inhibitors and two newly identified hits featuring a symmetric “Y”-shaped scaffold. Collectively, this 2C FP assay represents a robust and practical tool for quantifying the binding affinity and advancing the discovery of novel 2C allosteric inhibitors.

## Results and Discussion

### Structure-Based Design of the 2C FP Probe **Jun14157**

An unbound FP probe exhibits low polarization in the FP assay due to unrestricted rotation. Its motion is restricted upon binding to the 2C allosteric site, leading to a high polarization signal. A decrease in the polarization signal is observed if a testing compound competes for the same pocket and displaces the bound FP probe (Figure 2A).

**Figure 2:**
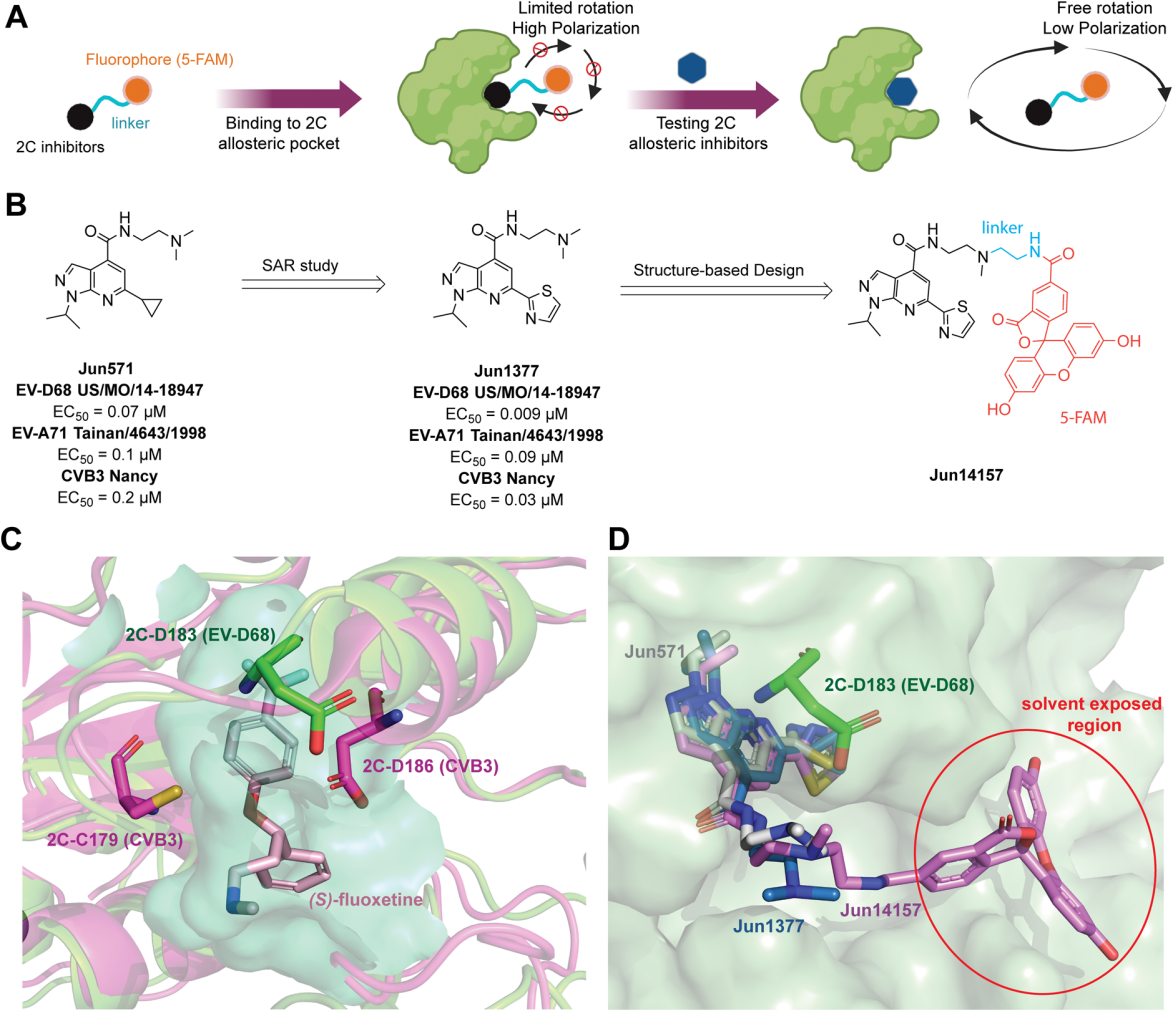
Design of the 2C FP probe. (A) FP assay principle. (B) SAR study and design of the 2C FP probe **Jun14157**. (C) Superimposed structures of EV-D68 2C (homology model) with CVB3 2C in complex with SFX (PDB ID 6T3W). EV-D68 2C is colored in green, CVB3 2C is colored in magenta, and the cavity of the allosteric pocket is colored in cyan. SFX is shown as wheat sticks. (D) The superposition of docking poses of **Jun571**, **Jun1377**, and **Jun14157** occurred at the allosteric site of EV-D68 2C. 2C is colored in split pea, **Jun571** is shown as gray sticks, **Jun1377** is shown as marine sticks, and **Jun14157** is shown as pink sticks.

Previously, we reported a series of pyrazolopyridine-containing inhibitors with broad-spectrum antiviral activity against enteroviruses, including the lead compound **Jun571** (Figure 2B).^27^ Resistance selection identified a 2C mutation, D183V, located at the SFX allosteric binding site, suggesting that **Jun571** binds to the same site as SFX (Figure 2C). Since the crystal structure of EV-D68 2C has not yet been determined, we built a homology model of EV-D68 US/MO/14-18947 2C using AlphaFold 3 (Figure S1 and Figure 2C).

Lead optimization on **Jun571** led to the discovery of **Jun1377**, featuring a thiazole-2-yl group at the 6-position, with low nanomolar EC_50_ values (Figure 2B). Notably, **Jun1377** demonstrated an approximately 8-fold increase in potency compared to **Jun571** against EV-D68 US/MO/14-18947 (0.009 μM vs 0.07 μM), EV-A71 Tainan/4643/1998 (0.09 μM vs 0.1 μM), and CVB3 Nancy (0.03 μM vs 0.2 μM) strains in cytopathic effect (CPE) assays (Figure 2B).

Next, we conducted molecular docking of **Jun571** and **Jun1377** in EV-D68 2C using Schrödinger Glide XP to guide the FP probe design. Both compounds showed superimposable binding poses with the dimethylamine group oriented toward the solvent-exposed region (Figure 2D). **Jun1377** exhibited a lower docking score (−8.069 kcal/mol) than **Jun571** (−7.782 kcal/mol), consistent with its superior antiviral activity. Given that the methyl group extends beyond the allosteric binding pocket, a short linker was deemed optimal (Figure 2D). Based on this, we designed and synthesized **Jun14157**, incorporating a two-carbon linker attached to 5-carboxyfluorescein (5-FAM) as a fluorophore (Figure 2B and Scheme 1). The predicted binding mode of **Jun14157** from molecular docking supports this design, with the fluorophore positioned on the 2C protein surface in a solvent-exposed region (Figure 2D).

**Scheme 1:**
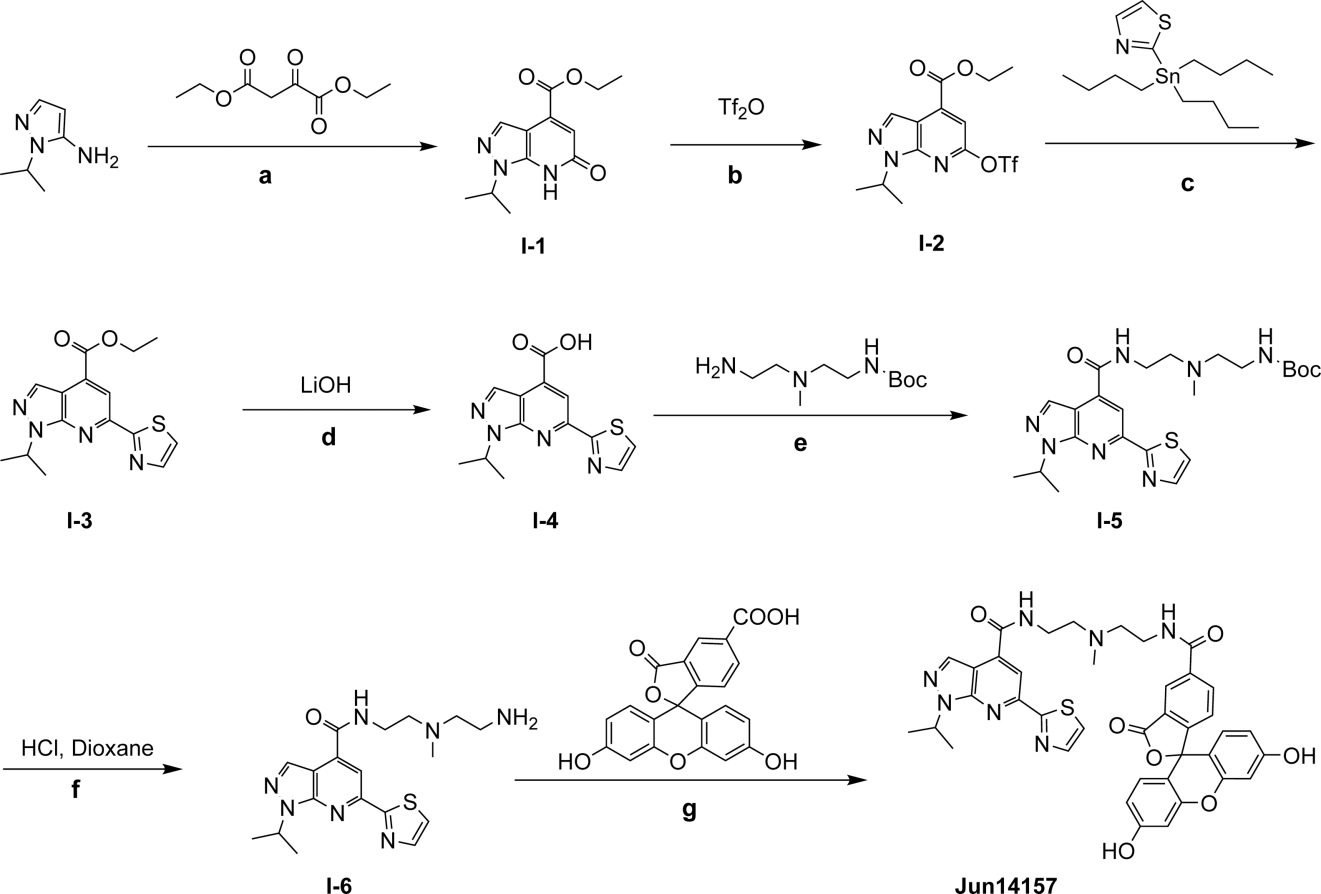
Synthetic Route of 2C FP Probe **Jun14157** ^a^

a Reaction conditions: (a) AcOH, water, reflux, 5 h; (b) pyridine, 0–25 °C, overnight; (c) Pd­(PPh_3_)_4_ (cat.), CsF, toluene, microwave, 115 °C, 75 min; (d) MeOH, water, 25 °C, overnight; (e) T_3_P, EtOAc, pyridine, 0–25 °C, overnight; (f) HCl, dioxane, DCM, 0–25 °C, 4 h; (g) HATU, DIPEA, DMF, 25 °C, overnight.

### Chemistry

The synthesis of FP probe **Jun14157** is shown in Scheme 1. Briefly, intermediate **I-1** was obtained through a one-step cyclization reaction. Next, **I-2** is synthesized by installing a triflyl group at the 6-position using trifluoromethanesulfonic anhydride (Tf_2_O). A subsequent Stille cross-coupling reaction introduced the 2-thiazolyl moiety at the 6-position to afford ethyl ester intermediate **I-3**. The ester was then hydrolyzed by using lithium hydroxide to yield **I-4**, which was subjected to the amide coupling reaction to attach the linker. The intermediate **I-5** underwent Boc deprotection and amide coupling with 5-carboxyfluorescein (5-FAM) to give the desired FP probe, **Jun14157**.

### 2C FP Assay Optimization

To optimize the FP assay, we first assessed the binding affinity between the tracer, **Jun14157**, and wild-type (WT) EV-D68 2C via a direct binding experiment. All FP assays were performed in a black 384-well flat-bottom plate with a 20 μL total volume following a previously reported “mix, incubate, and read” procedure.^37^ **Jun14157** was titrated at three different concentrations (20, 25, and 50 nM) with increasing concentrations of EV-D68 2C-WT in a HEPES buffer (50 mM HEPES, pH 7.5, 5 mM DTT, and 0.01% Triton X-100). After 30 min of incubation at room temperature (RT), the dose-dependent curves yielded similar dissociation constant (*K*_d_) values: 2.6 μM (20 nM tracer), 2.0 μM (25 nM tracer), and 2.6 μM (50 nM tracer). Based on the results, we selected 50 nM **Jun14157** and 5 μM EV-D68 2C-WT for further experiments, as this combination produced a favorable mP shift higher than 100 (ΔmP = 110) at the lowest 2C protein concentration (Figure 3A).

**Figure 3:**
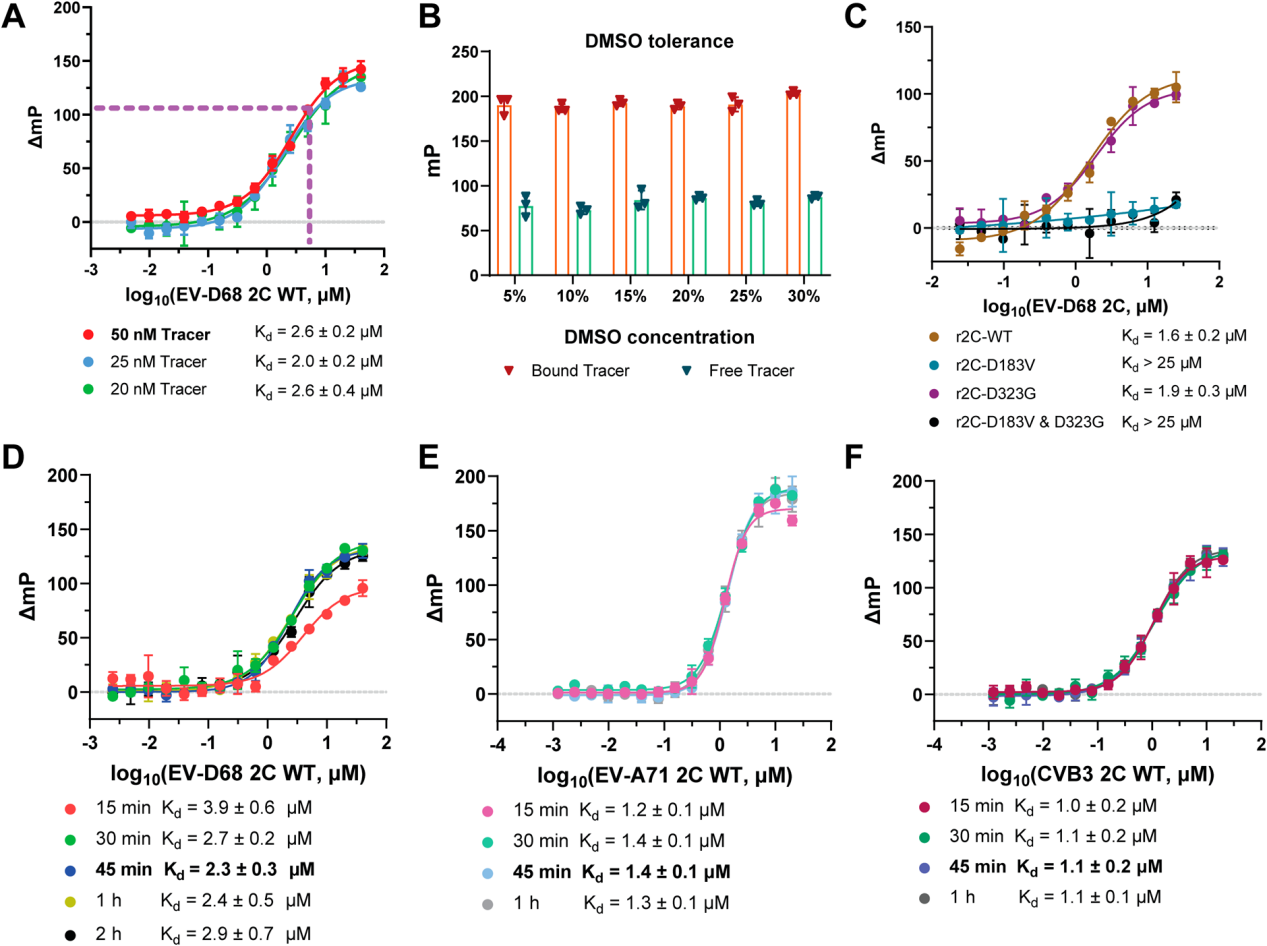
Optimization of the FP assay using the **Jun14157** probe. (A) Binding curves of the **Jun14157** probe at 20, 50, and 100 nM with increasing concentrations of EV-D68 2C after 30 min of incubation. *K*_d_ values represent means ± standard deviation (SD) from triplicate experiments. (B) Effect of dimethyl sulfoxide (DMSO) concentration on the mP values of bound and free probes. The assay was performed in triplicate. (C) Binding curves of 50 nM **Jun14157** with increasing concentrations of WT and EV-D68 2C mutants D183V, D323G, and D183V/D323G. *K*_d_ values are the mean ± SD from duplicate experiments. (D–F) FP titration curves of **Jun14157** with EV-D68 2C-WT (D), EV-A71 2C (E), and CVB3 2C (F) with different incubation times. *K*_d_ values are presented as the mean ± SD from triplicate experiments. Each data point represents the mean ± SD.

Next, we tested the dimethyl sulfoxide (DMSO) tolerance in the FP assay by evaluating polarization signals in buffers containing 5–30% DMSO using 50 nM **Jun14157** and 5 μM EV-D68 2C-WT. The polarization signal remained stable up to 30% DMSO after 30 min of incubation. For consistency, 10% DMSO (2 μL per 20 μL of assay) was chosen for subsequent experiments (Figure 3B).

With the optimized FP assay condition, we further validated **Jun14157**’s binding affinities to EV-D68 2C mutants D183V, D323G, and D183V/D323G.^27^ Previous studies have shown that D183V is resistant to **Jun571**, whereas D323G, located outside the allosteric site, remains sensitive.^27^ Titration of 50 nM **Jun14157** with these 2C proteins revealed that the probe bound to EV-D68 2C-WT (*K*_d_ = 1.6 μM) and D323G (*K*_d_ = 1.9 μM) with comparable binding affinities. In contrast, **Jun14157** had a reduced binding affinity to D183V-containing mutants (*K*_d_ > 25 μM for D183V and D183V/D323G) (Figure 3C). We also tested another 2C drug-resistant mutant F190L,^38^ which was also located in this allosteric drug binding pocket. It was found that EV-D68 2C-F190L similarly showed cross-resistance to **Jun14157** (*K*_d_ > 7.5 μM) (Figure S3). Overall, the results indicate that FP probe **Jun14157** is suitable for characterizing drug-resistant 2C mutants targeted by inhibitors that bind the same allosteric pocket as SFX.

To determine the optimal incubation time for the EV-D68 2C FP assay, we varied the preincubation time of the inhibitor with the 2C protein from 15 min to 2 h. Binding equilibrium was reached within 30 min, and the polarization signal (ΔmP) remained stable for up to 2 h. Based on these results, a 45 min incubation was selected for further experiments (Figure 3D).

We then extended the assay to other enteroviral 2C proteins by titrating **Jun14157** against EV-A71 2C and CVB3 2C proteins. The probe displayed higher affinities for EV-A71 2C (*K*_d_ = 1.4 μM) and CVB3 2C (*K*_d_ = 1.1 μM) compared to EV-D68 2C (*K*_d_ = 2.3 μM) (Figure 3E,F). A protein concentration of 2.5 μM was selected for both EV-A71 2C and CVB3 2C assays, as this condition yielded a favorable mP shift (ΔmP = 110). The maximum ΔmP is notably higher for EV-A71 2C compared to EV-D68 and CVB3 2Cs (Figure 3E vs Figure 3D,F), potentially reflecting greater protein stability and a higher proportion of properly folded protein.

Collectively, the optimized viral 2C FP assay conditions are 50 nM **Jun14157**, 5 μM for EV-D68 2C-WT or 2.5 μM for EV-A71 2C-WT and CVB3 2C-WT, HEPES buffer (50 mM HEPES, pH 7.5, 5 mM DTT, and 0.01% Triton X-100) with 10% DMSO, and 45 min of incubation before reading the polarization shift signal.

### Assay Validation with Reported 2C Inhibitors

To validate the 2C FP assay, 13 known 2C inhibitors, namely, guanidine, dibucaine, SFX, JX040, 6aw, 12a, **Jun571**, R523062, A967079, **Jun1377**, **Jun6504**, HBB, and pirlindole, were tested against the 2C proteins of EV-D68 US/MO/14-18947, EV-A71 Tainan/4643/1997, and CVB3 Nancy (Figures 1C, 2B, and 4A).^18,22^ Pleconaril (a VP1 capsid inhibitor) and telaprevir (a 2A protease inhibitor against EV-D68) were included as negative controls (Figure 4A).^39^ Additionally, the antiviral activities of all tested compounds against their respective viral strains were evaluated by using the CPE assay. The correlation between *K*_i_ values from the 2C FP assays and EC_50_ values from the CPE assay was plotted (Figure 4B). The detailed binding affinities and antiviral activity results are listed in Table S1.

**Figure 4:**
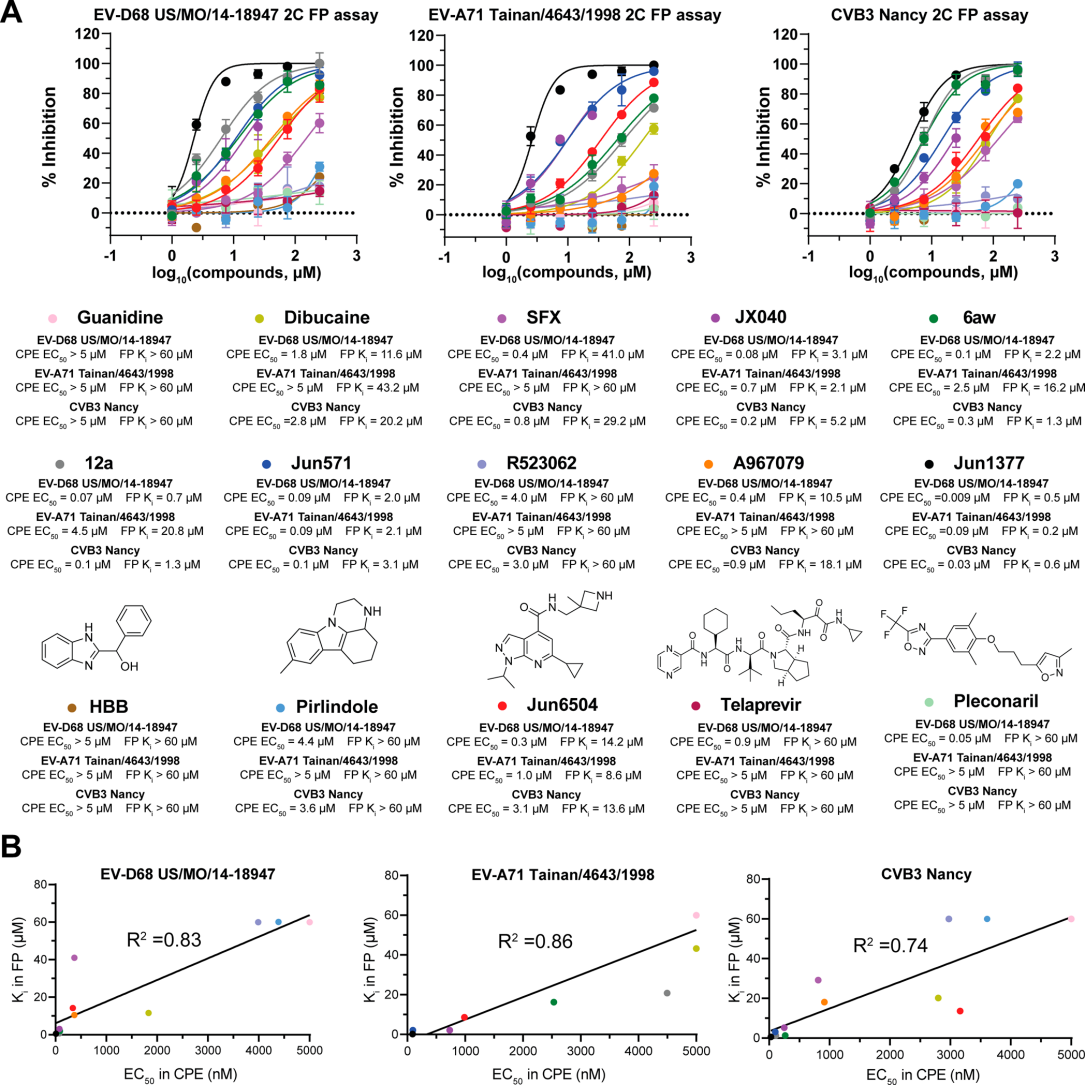
FP assay validation with reported 2C inhibitors and the correlation between the FP *K*_i_ values and the antiviral EC_50_ values for selected 2C inhibitors. (A) Competitive binding experiments measuring the displacement of the 2C FP probe **Jun14157** by guanidine, dibucaine, SFX, JX040, 6aw, 12a, **Jun571**, R523062, A967079, **Jun1377**, **Jun6504**, HBB, and pirlindole. The EV-D68 2A protease inhibitor (telaprevir) and capsid inhibitor (pleconaril) were used as negative controls. Plotted values from the FP assay are the mean ± SD (*n* = 2), and plotted values from the CPE assay are the mean ± SD (*n* = 3). (B) Correlation plot between the *K*_i_ values from the FP assay and the EC_50_ values from the CPE assay.

**Jun571**, **Jun1377**, and **Jun6504** showed consistent binding affinities across EV-D68, EV-A71, and CVB3 2C proteins (Figure 4A). In contrast, JX040, 6aw, and 12a displayed selective binding to EV-D68 and CVB3 2C, with weaker affinity for EV-A71 2C. Similarly, dibucaine, SFX, and A967079 bound to the allosteric site of EV-D68 and CVB3 2C but showed minimal interaction with EV-A71 2C. Guanidine, R523062, HBB, and pirlindole, along with the negative controls pleconaril and telaprevir, did not exhibit a dose-dependent response in the FP assays (Figure 4A). Although guanidine, R523062, HBB, and pirlindole were claimed as 2C inhibitors based on the resistance selection results,^26,28,40^ our FP assay results suggest that these compounds do not target the SFX-binding site and do not compete with the FP probe **Jun14157**.

A strong correlation was observed between the inhibition constant *K*_i_ values from the 2C FP assays and the antiviral EC_50_ values from the CPE assay, with *R* ^2^ values of 0.83, 0.86, and 0.74 for EV-D68, EV-A71, and CVB3, respectively (Figure 4B). For example, dibucaine showed an IC_50_ of 45.8 μM (*K*_i_ = 11.6 μM) in the EV-D68 2C FP assay and an IC_50_ of 85.1 μM (*K*_i_ = 20.2 μM) in the CVB3 2C FP assay, with EC_50_ values of 1.8 and 2.8 μM against EV-D68 and CVB3, respectively. In contrast, dibucaine exhibited an IC_50_ of 165 μM (*K*_i_ = 43.2 μM) in the EV-A71 2C FP assay and showed no antiviral activity against EV-A71 at concentrations up to 5 μM. Overall, these results suggest that the *K*_i_ values from the 2C FP assay can be used to predict the antiviral activity EC_50_ values in cell-based antiviral assays.

### Virtual Screening of 2C Inhibitors Targeting the Allosteric Pocket

To demonstrate the utility of our 2C FP assay for high-throughput screening and hit validation, we implemented a structure-based virtual screening (SBVS) workflow to identify novel allosteric inhibitors of 2C protein (Figure 5A). Our previous structure–activity relationship (SAR) study of the pyrazolopyridine and quinoline-containing 2C inhibitors pointed out that the terminal tertiary protonated amine of the dimethylaminoethyl moiety is critical for the antiviral activity.^27,31,32^ We therefore generated a reaction-based combinatorial virtual library using the Enumerate Combinatorial Library module in DataWarrior (v06.04.02, OpenMolecules). A total of 200,000 virtual compounds were generated in silico via an amide bond formation between 2-dimethylaminoethylamine and a diverse selection of commercially available aromatic carboxylic acids. The resulting library was filtered to retain compounds with a molecular weight of 190–400 Da and a calculated log *P* (cLogP) between 0 and 5, while excluding structural motifs commonly associated with nonspecific reactivity, cytotoxicity, or metabolic liability, such as esters, nitro groups, aldehydes, and Michael acceptors.

**Figure 5:**
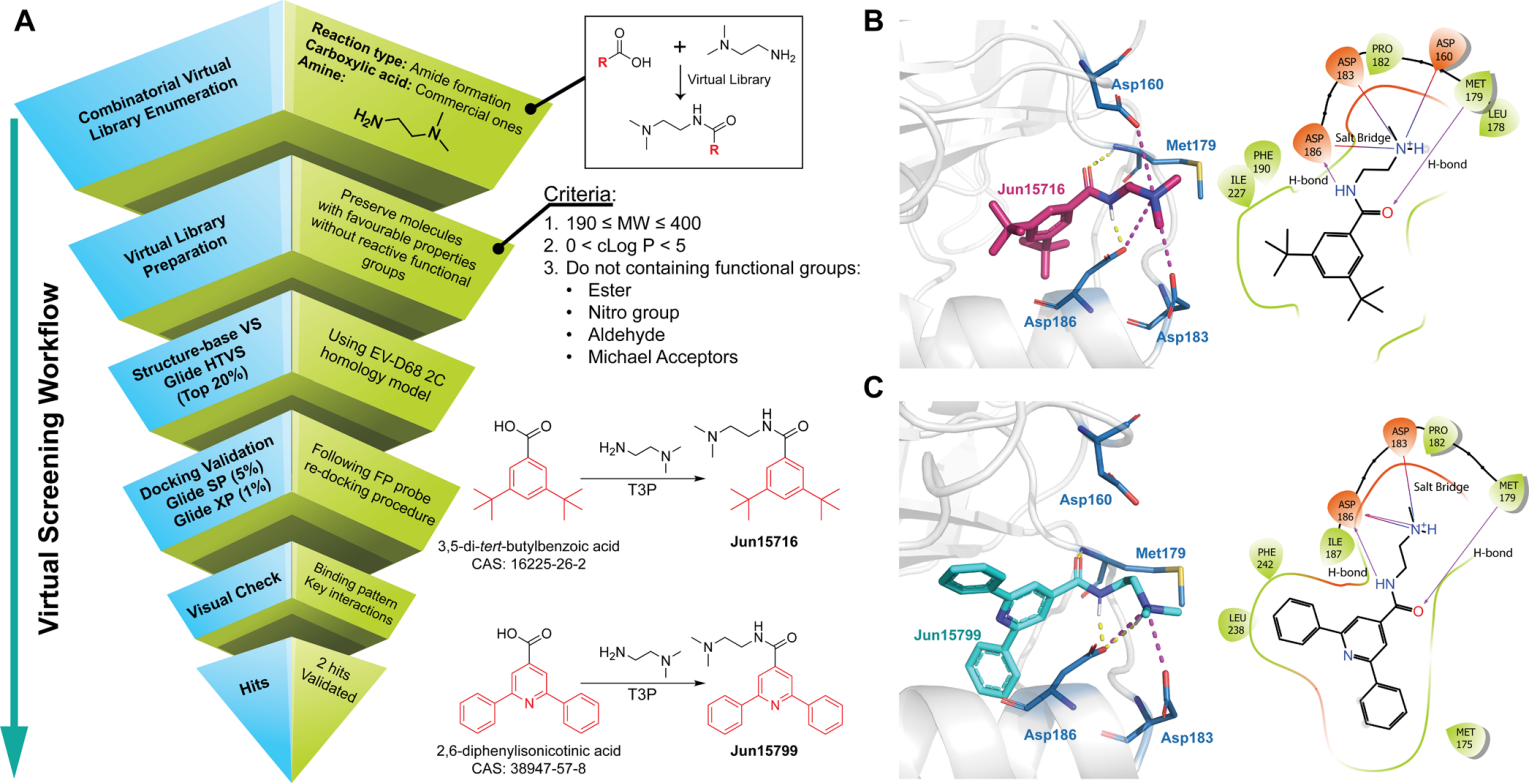
Virtual screening and hit validation of novel 2C inhibitors. (A) Graphical workflow of the virtual screening for identifying novel 2C inhibitors against enteroviruses. (B) Docking pose and 2D-binding pattern of **Jun15716**. (C) Docking pose and 2D-binding pattern of **Jun15799**. The H-bonds were shown as yellow dashed lines in docking poses and purple arrow lines in 2D-binding patterns. The salt bridges are shown as magenta dashed lines in docking poses and red-to-blue gradient lines in 2D-binding patterns.

Virtual screening was carried out using a homology model of EV-D68 2C generated by AlphaFold 3, employing Glide in high-throughput virtual screening (HTVS), standard precision (SP), and extra precision (XP) modes. The top 20% of scoring hits from the HTVS mode were preserved for the docking validation with Glide SP and XP. Top-scoring hits from the XP mode were visually inspected for key binding interactions with conserved residues (Figure S4). Two compounds, **Jun15716** and **Jun15799**, were selected for further validation based on their favorable docking poses (Figure 5B,C). Specifically, the carbonyl groups of both compounds form hydrogen bonds with the backbone NH of Met179, while their amide NH forms hydrogen bonds with the side chain carboxylate of Asp186. The terminal protonated dimethylamino group of **Jun15716** forms electrostatic interactions (salt bridges) with Asp160, Asp183, and Asp186 (Figure 5B), and the terminal protonated dimethylamino group of **Jun15799** interacts with Asp183 and Asp186 (Figure 5C).

To experimentally validate the SBVS hits, we synthesized **Jun15716** and **Jun15799** from 3,5-di-*tert*-butylbenzoic acid and 2,6-diphenylisonicotinic acid using a one-step amide coupling with T3P (Figure 5A).

Next, we validated two virtual screening hits, **Jun15716** and **Jun15799**, in dose–response FP assays against 2C proteins from EV-D68, EV-A71, and CVB3. **Jun15716** exhibited moderate binding affinity, with *K*_i_ values ranging from 15.9 to 44.2 μM, namely, 15.9 μM for EV-D68 2C, 17.8 μM for CVB3 2C, and 44.2 μM for EV-A71 2C (Figure 6A). In contrast, **Jun15799** displayed more potent binding affinities, with *K*_i_ values of 0.8 μM (EV-D68 2C), 3.0 μM (CVB3 2C), and 21.1 μM (EV-A71 2C) (Figure 6B).

**Figure 6:**
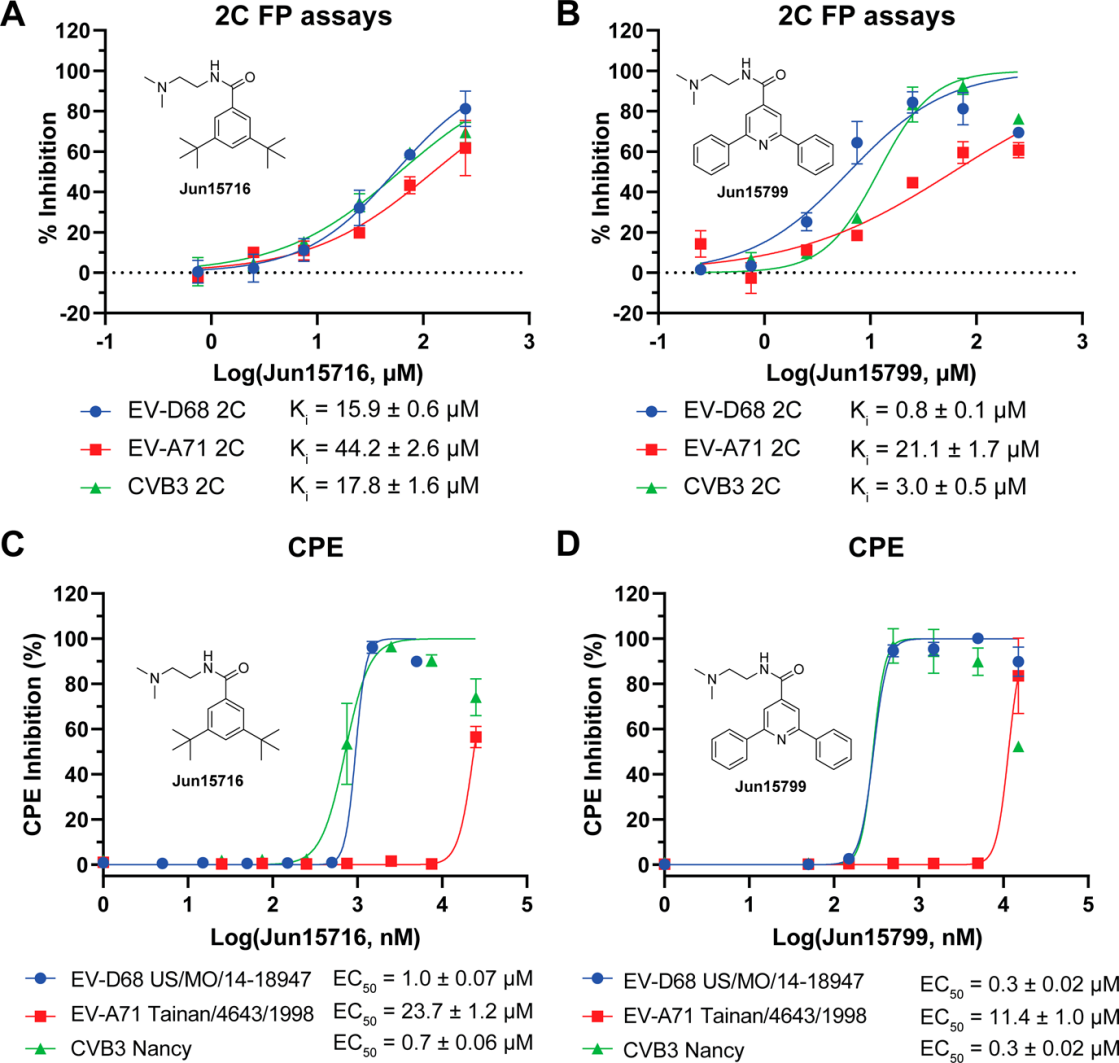
(A–D) Hit validation with the 2C FP and CPE assays. For the IC_50_ obtained from FP assays, the plotted values are the mean ± SD (*n* = 2). For EC_50_ obtained from CPE assays, the plotted values are the mean ± SD (*n* = 3).

To confirm the biochemical assay results, we evaluated their antiviral potency in a cell-based CPE assay. **Jun15716** inhibited EV-D68 US/MO/14-18947 and CVB3 Nancy with EC_50_ values of 1.0 and 0.7 μM, respectively, while it was less effective against EV-A71 Tainan/4643/1998 (EC_50_ = 23.7 μM) (Figure 6C). **Jun15799** showed more potent antiviral activity, with EC_50_ values of 0.3 μM (EV-D68), 0.3 μM (CVB3), and 11.4 μM (EV-A71) (Figure 6D). The detailed IC_50_, *K*_i_, and EC_50_ data are summarized in Table S1.

Together, these findings demonstrate the reliability of the 2C FP assay for inhibitor characterization and support the potential of **Jun15716** and **Jun15799** as novel “Y”-shaped symmetric 2C inhibitors for further optimization and development.

### Determination of the *Z*′ Factor for High-Throughput Screening

We evaluated the suitability of our 2C FP assay for high-throughput screening (HTS) by conducting a pilot experiment in a 384-well format using **Jun571** as a positive control. The assay yielded a signal-to-background ratio of 2.6 and a *Z*′ factor of 0.69 (Figure 7), indicating excellent assay performance for HTS. A *Z*′ factor between 0.5 and 1.0 is widely accepted as the benchmark for robust and reliable screening assays.^41^

**Figure 7:**
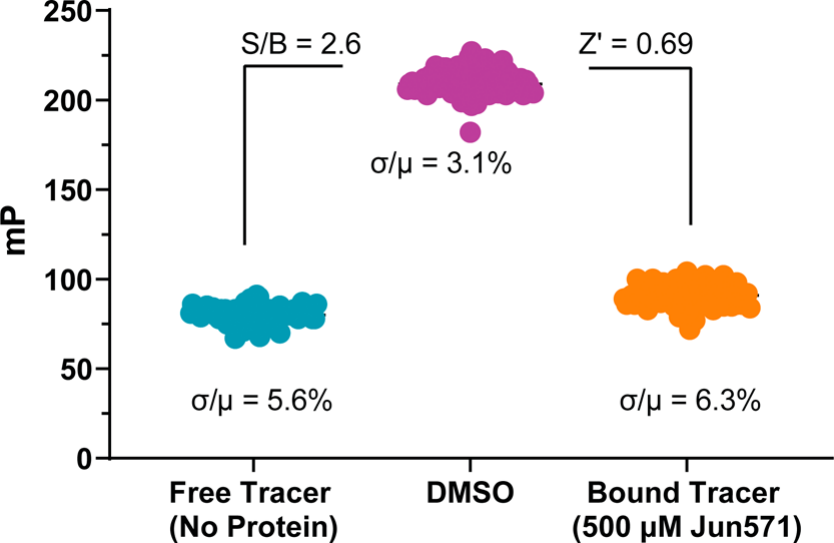
Determination of the *Z*′ factor in the FP assay. Five hundred μM **Jun571** and DMSO were treated as positive and negative controls, respectively. For each group, the data points were obtained in 96 replicates with a sample size (*n* = 96).

## Conclusions

The 2C protein remains a compelling antiviral target across diverse EV species. Several inhibitors have been shown to bind a conserved allosteric pocket adjacent to its ATP-binding site.^18,22^ However, a bottleneck in the field is the absence of a direct binding assay suitable for screening and characterizing allosteric 2C inhibitors. This study addressed this gap by developing and validating a robust, highly sensitive FP assay targeting the 2C allosteric site using a novel fluorescent probe, **Jun14157**. The FP assay was optimized for multiple EV 2C proteins, including those from EV-D68, EV-A71, and CVB3, demonstrating high reproducibility. Binding specificity was confirmed through site-directed mutagenesis and competition with known 2C inhibitors. Applying this assay to a panel of reported 2C inhibitors revealed a strong correlation with antiviral potencies in cell-based assays, underscoring the assay’s value in early stage hit validation. Interestingly, not all the reported 2C inhibitors target the SFX allosteric binding site, such as guanidine, R523062, and pirlindole. Moreover, virtual screening followed by FP-based validation demonstrated the potential of this assay in discovering novel 2C inhibitors targeting the conserved allosteric pocket. Notably, two “Y”-shaped hits, **Jun15716** and **Jun15799**, emerged as potent 2C inhibitors, warranting further optimization. Overall, this FP assay fills a critical methodological gap in enterovirus drug discovery by enabling the rapid and quantitative evaluation of compound binding, and it supports the rational design of next-generation 2C inhibitors. The platform established here for EV 2C proteins, leveraging a strategy of rational FP probe design and direct binding assay development, exemplifies a versatile approach that can be readily adapted to other therapeutically known drug targets.

## Experimental Section

### General Information

Solvents and commercially available building blocks were purchased from suppliers and used without purification. All reactions were monitored by thin-layer chromatography (TLC) visualization under ultraviolet light (254 nm) or liquid chromatography–mass spectrometry (LC-MS). Column chromatography purification was performed by using a CombiFlash NextGen 300+ system. High-performance liquid chromatography purification was performed by using an ACCQPrep HP150 system. The ESI-MS readings were recorded on an Agilent MSD iQ G6160A mass spectrometer. ^1^H NMR and ^13^C NMR spectra were recorded on a Bruker AV-400 spectrometer at 400 and 100 MHz, respectively. Coupling constants (*J*) are expressed in hertz (Hz). NMR data are analyzed with MestReNova (14.1.0). Chemical shifts (δ) of NMR are reported in parts per million (ppm) units. High-resolution mass spectra were recorded on a Q-TOF Premier mass spectrometer. The purity of the compounds was determined to be over 95% by reverse-phase HPLC analysis. All compounds were characterized by proton and carbon NMR and MS.

#### Ethyl 1-Isopropyl-6-oxo-6,7-dihydro-1*H*-pyrazolo­[3,4-*b*]­pyridine-4-carboxylate (**I-1**)

1-Isopropyl-1*H*-pyrazol-5-amine (1.24 g, 9.9 mmol) and diethyl 2-oxosuccinate (2.08 g, 9.9 mmol) were dissolved in 50 mL of acetic acid at room temperature. The reaction mixture was then refluxed for 5 h under a nitrogen atmosphere. After cooling to room temperature, the solvent was removed in vacuo, and the resulting residue was purified by silica gel flash column chromatography to give the intermediate **I-1** (1.24 g, 5.0 mM, 50% yield) as an orange solid. ^1^H NMR (400 MHz, DMSO-*d*_6_): δ 8.07 (s, 1H), 6.93 (s, 1H), 4.98 (p, *J* = 6.7 Hz, 1H), 4.39 (q, *J* = 7.1 Hz, 2H), 1.90 (s, 1H), 1.44 (d, *J* = 6.7 Hz, 6H), 1.37 (t, *J* = 7.1 Hz, 3H). ^13^C NMR (101 MHz, DMSO-*d*_6_): δ 164.7, 163.6, 134.3, 132.7, 62.1, 48.4, 22.3, 14.5. Chemical formula: C_12_H_15_N_3_O_3_; MS calculated for *m*/*z* [M + H]^+^: 250.1 (calculated), 250.1 (found).

#### Ethyl 1-Isopropyl-6-(((trifluoromethyl)­sulfonyl)­oxy)-1*H*-pyrazolo­[3,4-*b*]­pyridine-4-carboxylate (**I-2**)

The intermediate **I-1** (835 mg, 3.36 mmol) was dissolved with anhydrous pyridine and stirred at 0 °C. Trifluoromethanesulfonic anhydride (1040 mg, 3.68 mmol) was added dropwise, and the reaction mixture was stirred at room temperature overnight. Pyridine was removed under vacuum. The residue was dissolved in dichloromethane (DCM) and was washed with saturated aq. NaHCO_3_. The organic layer was washed with brine, dried with sodium sulfate, and concentrated under vacuum. The reaction mixture was then purified by flash chromatography to get the intermediate **I-2** (947 mg, 2.48 mmol, 74% yield) as a white solid. ^1^H NMR (400 MHz, CDCl_3_): δ 8.48 (s, 1H), 7.56 (s, 1H), 5.18 (p, *J* = 6.7 Hz, 1H), 4.54 (q, *J* = 7.2 Hz, 2H), 1.62 (d, *J* = 6.7 Hz, 6H), 1.50 (d, *J* = 14.3 Hz, 3H). ^13^C NMR (101 MHz, CDCl_3_): δ 163.4, 153.9, 147.6, 135.9, 132.9, 123.5, 120.3, 117.1, 113.9, 113.6, 108.9, 62.5, 50.2, 29.7, 21.7, 14.2. Chemical formula: C_13_H_14_F_3_N_3_O_5_S; MS calculated for *m*/*z* [M + H]^+^: 382.1 (calcd), 382.0 (found).

#### Ethyl 1-Isopropyl-6-(thiazol-2-yl)-1*H*-pyrazolo­[3,4-*b*]­pyridine-4-carboxylate­(**I-3**)

The intermediate **I-2** (622 mg, 1.62 mmol) was dissolved in toluene in a microwave vessel. 2-(Tributylstannyl)­thiazole (730 mg, 1.96 mmol), palladium-tetrakis­(triphenylphosphine) (282 mg, 0.2 mmol), and cesium fluoride (445 mg, 2.94 mmol) were subsequently added to the microwave vessel. After air purging with nitrogen, the vessel was capped and heated for 1 h 15 min at 130 °C. The reaction mixture was then washed with brine and concentrated under vacuum. The reaction mixture was then purified by flash chromatography to give intermediate **I-3** (436 mg, 1.38 mmol, 85% yield) as a slightly yellow solid. ^1^H NMR (400 MHz, DMSO-*d*_6_): δ 8.44 (d, *J* = 9.3 Hz, 2H), 8.08 (d, *J* = 3.1 Hz, 1H), 8.00 (d, *J* = 3.1 Hz, 1H), 5.28 (p, *J* = 6.7 Hz, 1H), 4.49 (q, *J* = 7.0 Hz, 2H), 1.58 (d, *J* = 6.6 Hz, 6H), 1.45 (t, *J* = 7.1 Hz, 3H). ^13^C NMR (101 MHz, DMSO-*d*_6_): δ 167.7, 164.5, 150.1, 150.0, 145.1, 132.7, 132.4, 124.3, 113.9, 113.7, 62.4, 49.4, 28.2, 26.7, 22.4, 14.5, 14.1. Chemical formula: C_15_H_16_N_4_O_2_S; MS calculated for *m*/*z* [M + H]^+^: 317.1 (calcd), 317.1 (found).

#### 1-Isopropyl-6-(thiazol-2-yl)-1*H*-pyrazolo­[3,4-*b*]­pyridine-4-carboxylic Acid­(**I-4**)

The intermediate **I-3** (406 mg, 1.28 mmol) and LiOH (215 mg, 5.12 mmol) were dissolved in CH_3_OH (4.5 mL) and H_2_O (1.5 mL), and the mixture was stirred at room temperature overnight. The solvent was removed in vacuo, and the resulting residue was diluted with 1 N HCl solution. The precipitate was collected by filtration to give intermediate **I-4**, which was directly set up for the next step.

#### *tert*-Butyl (2-((2-(1-Isopropyl-6-(thiazol-2-yl)-1*H*-pyrazolo­[3,4-*b*]­pyridine-4-carboxamido)­ethyl)­(methyl)­amino)­ethyl)­carbamate (**I-5**)

The intermediate **I-4** (345 mg, 1.2 mmol) and *tert*-butyl (2-((2-aminoethyl)­(methyl)­amino)­ethyl)­carbamate (261 mg, 1.2 mmol) were dissolved in a solution of ethyl acetate and pyridine (2:1), and the solution was stirred at 0 °C. Propyl phosphonic anhydride (T3P, 50% in EtOAc, 1.5 g, 2.4 mmol) was added dropwise, and the reaction mixture was stirred at room temperature overnight. Reaction conversion was monitored using LC-MS. The reaction mixture was extracted with ethyl acetate and aq. NaHCO_3_ three times. The organic layers were combined and washed with brine, dried with sodium sulfate, and concentrated under vacuum. The reaction mixture was then purified by flash chromatography to get the intermediate **I-5** (424 mg, 0.9 mmol, 75% yield) as a yellow oil. ^1^H NMR (400 MHz, CDCl_3_): δ 8.44 (s, 1H), 8.31 (s, 1H), 7.93 (d, *J* = 3.2 Hz, 1H), 7.51 (d, *J* = 3.2 Hz, 1H), 5.35 (p, *J* = 6.7 Hz, 1H), 4.41 (t, *J* = 8.0 Hz, 1H), 3.61 (dd, *J* = 10.5, 5.3 Hz, 3H), 3.24 (q, *J* = 6.0 Hz, 2H), 2.70 (t, *J* = 6.1 Hz, 2H), 2.58 (t, *J* = 6.2 Hz, 2H), 2.33 (s, 3H), 1.64 (d, *J* = 6.7 Hz, 6H), 1.26 (s, 9H). ^13^C NMR (101 MHz, CDCl_3_): δ 168.9, 165.1, 162.6, 160.6, 156.1, 149.9, 149.8, 144.0, 136.9, 132.3, 122.1, 113.8, 110.8, 64.8, 56.8, 56.3, 49.2, 41.8, 40.6, 37.5, 36.4, 31.4, 28.2, 22.0. Chemical formula: C_23_H_33_N_7_O_3_S; MS calculated for *m*/*z* [M + H]^+^: 488.2 (calculated), 488.2 (found).

#### *N*-(2-((2-Aminoethyl)­(methyl)­amino)­ethyl)-1-isopropyl-6-(thiazol-2-yl)-1*H*-pyrazolo­[3,4-*b*]­pyridine-4-carboxamide (**I-6**)

Boc-protected intermediate **I-5** (438 mg, 0.9 mmol) was dissolved in DCM and stirred at 0 °C. Four M hydrogen chloride in dioxane solution (900 μL, 3.48 mmol) was added, and the reaction was stirred at room temperature for 2 h. The reaction was monitored by using LC-MS. The reaction mixture was extracted with DCM and sodium hydroxide (1 M). The organic layer was washed with brine, dried with sodium sulfate, and concentrated under vacuum. The resulting crude product **I-6** was directly set up for the next step.

#### *N*-(2-((2-(3′,6′-Dihydroxy-3-oxo-3*H*-spiro­[isobenzofuran-1,9′-xanthene]-5-carboxamido)­ethyl)­(methyl)­amino)­ethyl)-1-isopropyl-6-(thiazol-2-yl)-1*H*-pyrazolo­[3,4-*b*]­pyridine-4-carboxamide (**Jun14157**)

At room temperature, 5-carboxyfluorescein (350 mg, 0.9 mmol) was dissolved in 5 mL of anhydrous DMSO, HATU (410.6 mg, 1.08 mmol), and DIPEA (349 mg, 2.7 mmol), and the reaction mixture was stirred for 30 min under nitrogen protection; then, intermediate **I-6** (335 mg, 0.9 mmol) was added and continued to stir for 12 h. The reaction mixture was concentrated and dried under vacuum. The final compounds were purified with Prep-HPLC to provide desired product **Jun14157** (75 mg, 0.1 mmol, 11% yield) as a yellow solid. ^1^H NMR (400 MHz, DMSO-*d*_6_): δ 9.37 (t, *J* = 5.6 Hz, 1H), 9.16 (t, *J* = 5.6 Hz, 1H), 8.50 (d, *J* = 2.6 Hz, 3H), 8.22 (dd, *J* = 8.0, 1.5 Hz, 1H), 8.10 (d, *J* = 3.2 Hz, 1H), 8.01 (d, *J* = 3.2 Hz, 1H), 7.33 (d, *J* = 8.0 Hz, 1H), 6.77 (d, *J* = 1.5 Hz, 2H), 6.60 (s, 4H), 5.30 (p, *J* = 6.6 Hz, 1H), 3.83 (dq, *J* = 18.4, 5.9 Hz, 4H), 3.69–3.59 (m, 2H), 3.50 (d, *J* = 16.4 Hz, 2H), 3.08 (s, 3H), 1.58 (d, *J* = 6.7 Hz, 6H). ^13^C NMR (101 MHz, DMSO-*d*_6_): δ 168.5, 168.1, 166.0, 165.5, 160.2, 159.1, 158.7, 155.5, 152.3, 150.0, 149.8, 144.8, 137.1, 136.0, 135.2, 132.9, 129.4, 126.9, 124.6, 124.2, 123.9, 117.6, 114.7, 113.8, 113.2, 111.5, 109.4, 102.8, 83.9, 55.0, 54.8, 49.2, 35.0, 34.9, 22.3. Chemical formula: C_39_H_35_N_7_O_7_S; HRMS calculated for *m*/*z* [M + H]^+^: 746.2397 (calculated), 746.2391 (found).

#### *N*-(2-(Dimethylamino)­ethyl)-1-isopropyl-6-(thiazol-2-yl)-1*H*-pyrazolo­[3,4-*b*]­pyridine-4-carboxamide (**Jun1377**)

The intermediate **I-4** (100 mg, 0.35 mmol) and *N*,*N-*dimethylethylenediamine (50 mg, 0.38 mmol) were dissolved in a solution of ethyl acetate and pyridine (2:1), and the solution was stirred at 0 °C. Propyl phosphonic anhydride (T3P, 50% in EtOAc, 445 mg, 0.7 mmol) was added dropwise, and the reaction mixture was stirred at room temperature overnight. Reaction conversion was monitored using LC-MS. The reaction mixture was extracted with ethyl acetate and aq. NaHCO_3_ three times. The organic layers were combined and washed with brine, dried with sodium sulfate, and concentrated under vacuum. The reaction mixture was then purified by flash chromatography to get **Jun1377** (80 mg, 0.25 mmol, 72% yield) as a yellow solid. ^1^H NMR (400 MHz, DMSO-*d*_6_): δ 9.25 (t, *J* = 5.7 Hz, 1H), 8.44 (d, *J* = 3.3 Hz, 2H), 8.09 (d, *J* = 3.4 Hz, 1H), 8.00 (d, *J* = 3.1 Hz, 1H), 5.26 (dt, *J* = 13.8, 6.8 Hz, 1H), 3.71 (d, *J* = 6.4 Hz, 2H), 3.39–3.32 (m, 2H), 2.88 (s, 6H), 1.56 (d, *J* = 6.5 Hz, 6H). ^13^C NMR (101 MHz, DMSO-*d*_6_): δ 168.2, 165.4, 150.0, 149.9, 144.9, 137.2, 133.0, 124.3, 113.9, 111.5, 56.2, 49.2, 43.0, 35.2, 22.4. Chemical formula: C_17_H_22_N_6_OS; HRMS calculated for *m*/*z* [M + H]^+^: 359.1654 (calculated), 359.1645 (found).

#### 3,5-Di-*tert*-butyl-*N*-(2-(dimethylamino)­ethyl)­benzamide (**Jun15716**)

The 3,5-di-*tert*-butylbenzoic acid (200 mg, 0.85 mmol) and *N,N-*dimethylethylenediamine (83 mg, 0.94 mmol) were dissolved in a solution of ethyl acetate and pyridine (2:1), and the solution was stirred at 0 °C. Propyl phosphonic anhydride (T3P, 50% in EtOAc, 540 mg, 1.7 mmol) was added dropwise, and the reaction mixture was stirred at room temperature overnight. Reaction conversion was monitored using LC-MS. The reaction mixture was extracted with ethyl acetate and aq. NaHCO_3_ three times. The organic layers were combined and washed with brine, dried with sodium sulfate, and concentrated under vacuum. The reaction mixture was then purified by flash chromatography to get **Jun15716** (185 mg, 0.6 mmol, 71% yield) as a white solid. ^1^H NMR (400 MHz, CDCl_3_): δ 8.49 (d, *J* = 6.0 Hz, 1H), 7.66 (d, *J* = 1.9 Hz, 2H), 7.59 (t, *J* = 1.8 Hz, 1H), 3.85 (q, *J* = 5.6 Hz, 2H), 3.38 (q, *J* = 5.5, 5.1 Hz, 2H), 2.91 (d, *J* = 3.5 Hz, 6H), 1.30 (s, 16H). ^13^C NMR (101 MHz, CDCl_3_): δ 170.0, 151.4, 132.0, 126.6, 121.5, 58.4, 44.1, 35.6, 35.0, 31.2. NMR (101 MHz, CDCl_3_): δ 170.0, 151.5, 131.9, 126.6, 121.6, 58.4, 44.1, 35.6, 34.9, 31.2. Chemical formula: C_19_H_32_N_2_O; HRMS calculated for *m*/*z* [M + H]^+^: 305.2593 (calculated), 305.2573 (found).

#### *N*-(2-(Dimethylamino)­ethyl)-2,6-diphenylisonicotinamide (**Jun15799**)

The 2,6-diphenylisonicotinic acid (275 mg, 1 mmol) and *N,N-*dimethylethylenediamine (97 mg, 1.1 mmol) were dissolved in a solution of ethyl acetate and pyridine (2:1), and the solution was stirred at 0 °C. Propyl phosphonic anhydride (T3P, 50% in EtOAc, 1.27 g, 2 mmol) was added dropwise, and the reaction mixture was stirred at room temperature overnight. Reaction conversion was monitored using LC-MS. The reaction mixture was extracted with ethyl acetate and aq. NaHCO_3_ three times. The organic layers were combined and washed with brine, dried with sodium sulfate, and concentrated under vacuum. The reaction mixture was then purified by flash chromatography to get **Jun15799** (214 mg, 0.62 mmol, 62% yield) as a white solid. ^1^H NMR (400 MHz, CDCl_3_): δ 9.07 (t, *J* = 5.7 Hz, 1H), 8.13 (s, 2H), 8.04 (dd, *J* = 6.5, 2.8 Hz, 4H), 7.46 (dd, *J* = 5.3, 1.9 Hz, 6H), 7.32 (s, 2H), 3.79 (q, *J* = 5.5 Hz, 2H), 3.28 (t, *J* = 5.4 Hz, 2H), 2.82 (s, 6H). ^13^C NMR (101 MHz, CDCl_3_): δ 167.0, 157.8, 142.2, 138.1, 129.7, 128.8, 127.2, 116.5, 58.1, 44.0, 35.4. Chemical formula: C_22_H_23_N_3_O; HRMS calculated for *m*/*z* [M + H]^+^: 346.1919 (calculated), 346.1901 (found).

### Reagents

Unless otherwise noted, all biological reagents, control compounds, and consumables were purchased from commercial vendors.

### EV-D68 2C (40–330) WT and Mutant Protein Expression

The EV-D68 US/MO/14-18947 2C (amino acid residues 40–330), including WT and mutant proteins (2C-D183V, 2C-F190L, 2C-D323G, and 2C-D183V/D323G), were expressed and purified modified from a previously reported protocol.^28^ Briefly, a codon-optimized DNA fragment encoding EV-D68 2C (residues 40–330) was synthesized by GenScript (Piscataway, NJ) and cloned into the pET28a­(+)-TEV expression vector. Site-directed mutagenesis (D183V, F190L, D323G, and D183V/D323G) was performed by using a QuikChange XL kit (Agilent, Santa Clara, CA). The recombinant plasmids were transformed into () BL21 (DE3) competent cells. Cultures were grown in LB medium containing 50 μg/mL kanamycin at 37 °C to an optical density at 600 nm (OD_600_) of 0.6–0.8; then, the protein expression was induced with 0.5 mM isopropyl β-d-1-thiogalactopyranoside (IPTG). Postinduction, cultures were incubated at 18 °C for 12–16 h. Cells were harvested by centrifugation (7500*g*, 5 min, 4 °C), resuspended in lysis buffer (20 mM HEPES, pH 7.5; 300 mM NaCl; 4 mg/mL lysozyme; 1 mM PMSF; 0.01 mg/mL DNase I), and lysed by sonication for 30 min. The lysate was clarified by centrifugation at 17,000*g* for 1 h at 4 °C. The resulting supernatant was incubated overnight with Ni-NTA resin at 4 °C. Bound proteins were eluted using a gradient of imidazole and further purified to >98% homogeneity, followed by buffer exchange using FPLC desalting columns (HiPrep 26/10 desalting, lot no. 308273) with buffer (20 mM HEPES, pH 7.5; 300 mM NaCl; 1 mM DTT). Final protein preparations were flash-frozen in liquid nitrogen and stored at −80 °C.

### EV-A71 2C (40–329) WT and CVB3 2C (37–329) WT Protein Expression

EV-A71 Tainan/4643/1998 2C (amino acid residues 40–329) protein and CVB3 Nancy 2C (amino acids 37–329) protein were expressed and purified as described.^32^ Similarly, codon-optimized DNA fragments were synthesized by GenScript (Piscataway, NJ) and cloned into the pET28a (+)-TEV vector. Recombinant plasmids were transformed into Rosetta 2­(DE3) competent cells. Cultures were grown in LB medium supplemented with 50 μg/mL kanamycin and 34 μg/mL chloramphenicol. Expression and lysis procedures were identical to those used for EV-D68 2C expression. After clarification, supernatants were incubated with Ni-NTA resin for over 2 h at 4 °C. Proteins were eluted with imidazole and purified to >98% purity and then dialyzed for 4 h twice against buffer (20 mM HEPES, pH 7.5; 300 mM NaCl; 1 mM DTT). Final protein samples were flash-frozen in liquid nitrogen and stored at −80 °C.

### Molecular Docking

All of the protein complex structures were processed with the Schrödinger Protein Preparation Wizard. The docking was performed using Schrödinger Glide with extra precision (XP). The docking grid was centered around SFX. **Jun571**, **Jun1377**, **Jun14157**, **Jun15716**, and **Jun15799** were prepared and docked following the generic procedure of the Ligand Docking module. The final docking poses were illustrated in PyMOL.

### Virtual Library Enumeration, Preparation, and Virtual Screening

A reaction-based combinatorial virtual library was generated using the Enumerate Combinatorial Library module in DataWarrior (v06.04.02, OpenMolecules), with amide bond formation selected as the reaction type. 2-Dimethylaminoethylamine was used as the sole amine reactant, while the number of commercially available carboxylic acid reactants was set to 200,000 from different vendors. For all enumerated compounds, key physicochemical properties, including molecular weight (MW) and calculated log *P* (cLogP), were computed. R-group decomposition was also performed to enable further substructure analysis. Compounds with MW <190 or >400, cLogP <0 or >5, or containing potentially reactive or unstable functional groups, including esters, nitro groups, aldehydes, and Michael acceptors, were excluded. After this filtering step, approximately 15,000 compounds remained. These were exported in an SDF format and subjected to ligand preparation using the LigPrep module. Virtual screening was then conducted using Glide in a hierarchical docking workflow: HTVS mode was first applied to the full library, retaining the top 20% of scoring compounds; these were further refined using SP mode (top 2% retained), followed by XP mode (top 1%, yielding ∼500 compounds), which were selected for manual inspection based on binding pose and key interactions.

### Direct Binding Assay for 2C Titration

To develop the FP assay for HTS, all of the experiments were performed in 384-well, flat-bottom, black assay plates (3821 Costar, Corning) at a final volume of 20 μL. The assay buffer contains 50 mM HEPES (pH 7.5), 0.01% Triton X-100, and 5 mM DTT. A 2-fold dilution for purified 2C proteins was carried out in the assay buffer. Automation of solution handling and tracer addition was then performed to add 18 μL of diluted 2C solution and 2 μL of **Jun14157** in DMSO at 10× desired concentrations. Unless otherwise specified, the final DMSO concentration was 10%, and all plates measured in FP plates were incubated and shaken for 30 min at RT. The polarization was measured using a Cytation 5 equipped with an FP filter cube (8040561, Agilent, ex: 485/20, em: 528/20). The mP values were calculated using the equation below:

$$\mathrm{mP}=\frac{F_{∥}-G\times F_{⊥}}{F_{∥}-G\times F_{⊥}}\times 1000$$

where *F*_∥_ and *F*_⊥_ are the parallel and perpendicular fluorescence intensities, respectively, and *G* is the grating factor of the instrument. The ΔmP value was calculated using the equation below:

$$\Delta \mathrm{mP}={\mathrm{mP}}_{\mathrm{test}}-{\mathrm{mP}}_{\mathrm{tracer}}$$

where mP_test_ and mP_tracer_ are the mP values of the test wells and free tracer control (no protein) wells, respectively. The obtained ΔmP values and 2C concentrations were fitted in the one-site-specific binding model implemented in GraphPad 9 to give the *K*_d_ values.

### Equilibrium Binding Experiment for FP Signal Stability Determination

The equilibrium binding experiment was performed similarly to the direct binding assay, where 50 nM **Jun14157** (final concentration) and 2C (2-fold serial dilution). The equilibration time and signal stability were determined by monitoring the FP signal at different times. Between each reading, plates were covered with aluminum plate seals to prevent photobleaching and placed on a slow-moving shaker at RT until the next reading.

### Dimethyl Sulfoxide Tolerance

Six different assay buffers with an increasing concentration of DMSO in the assay buffer (0, 5, 10, 15, 20, and 25%) were used to dilute EV-D68 2C. Then, 1 μL of 1 μM **Jun14157** in DMSO was added to 19 μL of the diluted EV-D68 2C solution, resulting in the final total DMSO concentration ranging from 5 to 30% and the final EV-D68 2C concentration.

### General Competitive Binding Assay for Compound Testing

The procedure for the general competitive binding assay was divided into three steps, namely, mixing, incubation, and reading.^37^ Briefly, the 2C protein in the assay buffer was mixed with 1 μM **Jun14157** in DMSO with a volume ratio of 18:1 to give the 2C-tracer mixture. To each well, 19 μL of the mixture was added, followed by 1 μL of the testing compound in DMSO at a 20× desired concentration to reach the final volume of 20 μL. For compound testing, the compounds were 3-fold serially diluted using DMSO and tested in duplicate. The FP values were measured after a 45 min incubation on a slow-moving shaker. To normalize the results, the ΔmP value was calculated. The obtained data were fitted into an IC_50_ curve in GraphPad Prism. The *K*_i_ values were calculated following the equation with the IC_50_ values:^42^

$$K_{i}=\frac{{\left[I\right]}_{50}}{\left(\frac{{\left[L\right]}_{50}}{K_{d}}+\frac{{\left[P\right]}_{0}}{K_{d}}+1\right)}$$

where [I]_50_ and [L]_50_ are free inhibitor concentration and free tracer concentration at 50% inhibition, [P]_0_ is free protein concentration at 0% inhibition, and *K*_d_ is the dissociation constant between 2C and the testing compound. [I]_50_ and [L]_50_ were calculated following the equations in the reported literature.^43^

### *Z*′ Factor Statistical Experiments for Assay Accuracy and Precision Determination

*Z*′ factor statistical experiments were performed with three groups: a negative control group (50 nM **Jun14157** bound to EV-D68 2C), a positive control group (50 nM **Jun14157** displaced from EV-D68 2C by **Jun571** at 500 μM) in 96 replicates, and a background group (no protein). The *Z*′ factor was calculated using the equation below:

$$Z^{\prime }=1-\frac{3\left(\sigma_{P}+\sigma_{N}\right)}{\left(\mu_{P}-\mu_{N}\right)}$$

where σ_P_ and σ_N_ are the standard deviations of ΔmP values of positive control and negative control wells, while μ_P_ and μ_N_ are the mean ΔmP values of positive control and negative control wells.

### Cell Lines and Viruses

Rhabdomyosarcoma (RD, ATCC, and CCL-136) and Vero (ATCC and CRL-81) were maintained in a 37 °C incubator under a 5% CO_2_ atmosphere. RD cells and Vero cells were cultured in Dulbecco’s modified Eagle’s medium (DMEM) with 10% fetal bovine serum (FBS) and 1% penicillin–streptomycin (P/S). The enterovirus strains used in this study were obtained from commercial sources: EV-D68 US/MO/14-18947 (ATCC, NR-49129), EV-A71 Tainan/4643/1998 (BEI Resources, NR-471), and CVB3 strain Nancy (ATCC, VR-30). EV-D68 and EV-A71 were amplified in RD cells, and CVB3 was amplified in Vero cells before infection assays.

### CPE Assays

CPE assays were carried out as previously described.^27^ For EV-D68 CPE assays, RD cells were grown to over 90% confluency after seeding in a 96-well plate for 18–24 h, the growth medium was aspirated, and cells were washed with 100 μL of PBS buffer. Cells were infected with EV-D68 viruses diluted in DMEM with 2% FBS and 30 mM MgCl_2_ at an MOI of 0.01 and incubated in a 33 °C incubator in a 5% CO_2_ atmosphere for 1–2 h. Testing compounds diluted in DMEM with 2% FBS and 30 mM MgCl_2_ were added, and cells were incubated in a 33 °C incubator with a 5% CO_2_ atmosphere for 3 days to develop complete CPE in virus-infected cells. For EV-A71 CPE assays, similar procedures were performed on RD cells except that 30 mM MgCl_2_ was not included in the medium, and infected cells were incubated at 37 °C instead of 33 °C. The incubation time was 2.5 days for EV-A71 virus to develop complete CPE. For the CVB3 virus CPE assay, Vero cells were used for infection with CVB3 Nancy virus at an MOI of 0.3, with a similar procedure as EV-A71. For all CPE assays, the growth media was aspirated, and 50 μg/mL neutral red staining solution was used to stain viable cells in each well. Absorbance at 540 nm was measured using a Multiskan FC microplate photometer (Thermo Fisher Scientific). The EC_50_ values were calculated from best-fit dose–response curves using GraphPad Prism 8.

## Supplementary Material





## Notes

Figures S1–S4, Table S1, HNMR and CNMR spectra for intermediates, and HPLC traces of the final products (PDF)
Molecular formula strings (CSV)

## Abbreviations

EV: Enterovirus
CVB3: Coxsackievirus B3
5-FAM: 5-carboxyfluorescein
FP: fluorescence polarization
PDB: Protein Data Bank
HTS: high-throughput screening
HTVS: high-throughput virtual screening
SP: standard precision
XP: extra precision
ORF: open reading frame
UTR: untranslated regions
ATP: adenosine triphosphate
DSF: differential scanning fluorimetry
TSA: thermal shift assay
SFX: (*S*)-fluoxetine
DTT: dithiothreitol
DMSO: dimethyl sulfoxide
H-bond: hydrogen bond
HBB: 2-(α-hydroxybenzyl)-benzimidazole
