# Supplementary material for: Design of a Fluorescence Polarization Probe for Enterovirus 2C Proteins
Source: J Med Chem. 2025 Jun 21;68(13):14041–53. doi: 10.1021/acs.jmedchem.5c01219 (PMC12333363; doi:10.1021/acs.jmedchem.5c01219)
Supplement: Supplementary file 1 [file jm5c01219_si_001.pdf]

Supplementary information

Design of a Fluorescence Polarization Probe for Enterovirus 2C Proteins

Kan Li[1], Hiwot A Demssie[1], Jun Wang[1,*]

[1]Department of Medicinal Chemistry, Ernest Mario School of Pharmacy, Rutgers, the State University of New Jersey, Piscataway, NJ, 08854, USA

*Corresponding author. Email: junwang@pharmacy.rutgers.edu (J.W.),

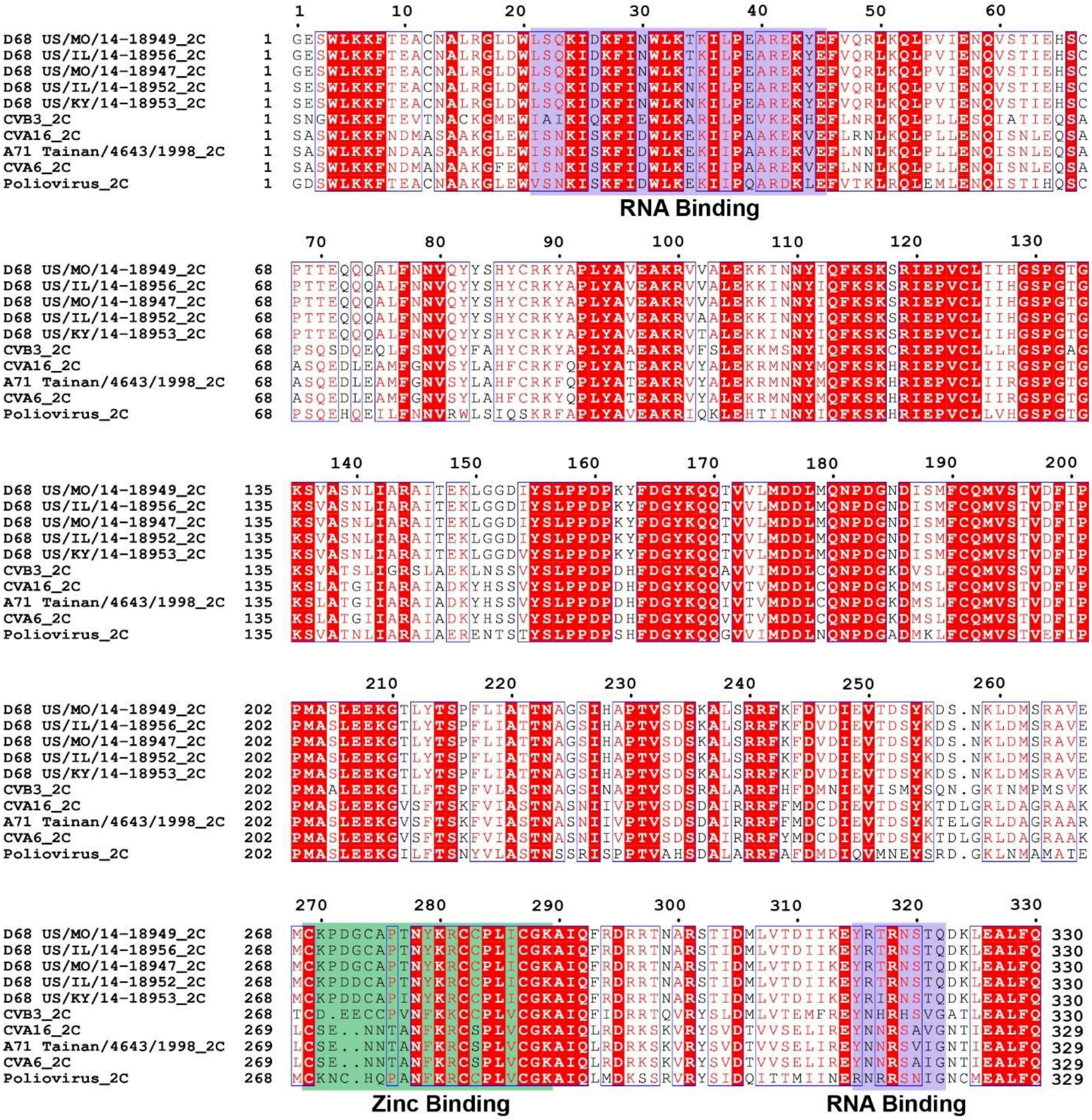

Figure S1. Sequence alignment of enterovirus 2C proteins. All sequences were downloaded from the NCBI genebank, the alignment analysis was performed using ClustalX 2.1, and the figure of the results was generated on ESPript 3 (https://espript.ibcp.fr/ESPript/cgi-bin/ESPript.cgi).

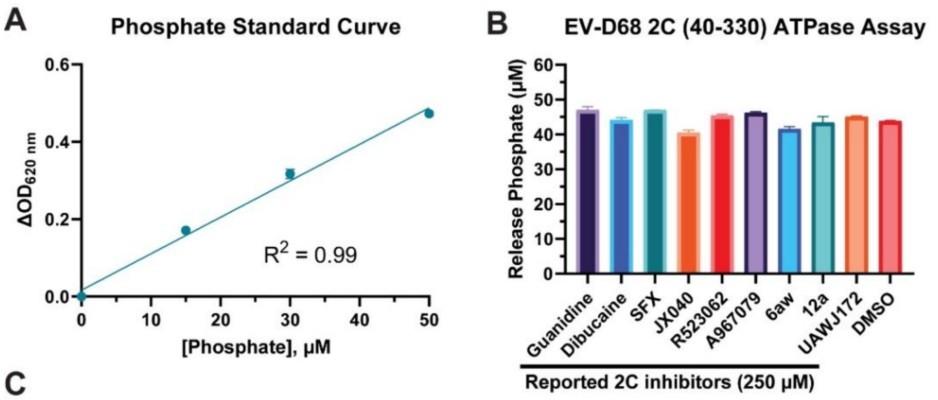

C

CVB3 2C (37-329)

H_3N^+-MGSSHHHHHHSSGENLYFQGHMLPEVREKHEFLNRLKQLPLLESQ
IATIEQSAPSQSDQEQLFSNVQYFAHYCRKYAPLYAAEAKRVFSLEKKMS
NYIQFKSKCRIEPVCLLLHGSPGAGKSVATNLIGRSLAEKLNSSVYSLPPD
PDHFDGYKQQAVVIMDDLCQNPDGKDVSLFCQMVSSVDFVPPMAALEE
KGILFTSPFVLASTNAGSINAPTVSDSRALARRFHFDMNIEVISMYSQNGK
INMPMSVKTCDDECCPVNFKKCCPLVCGKAIQFXDRRTQVRYSLXMLVT
EMXXSXTIVIAXGTTLXXLFQ*-COO^-

EV-D68 2C (40-330)

H_3N^+-MGSSHHHHHHSSGENLYFQGHMAREKYEFVQRLKQLPVIENQVST
IEHSCPTTEQQQALFNNVQYYSHYCRKYAPLYAVEAKRVVALEKKINNYIQ
FKSKSRIEPVCLIIHGSPGTGKSVASNLIARAITEKLGGDIYSLPPDPKYFD
GYKQQTVVLMDDLMQNPDGNDISMFCQMVSTVDFIPPMASLEEKGTLYT
SPFLIATTNAGSIHAPTVSDSKALSRRFKFDVDIEVTDSYKDSNKLDMSRA
VEMCKPDGCAPTNYKRCCPLICGKAIQFRDRRTNARSTIDMLVTDIIKEYR
TRNSTQDKLEALFQ*-COO^-

EV-A71 2C (40-329)

H_3N^+-MGSSHHHHHHSSGENLYFQGHMAKEKVEFLNNLKQLPLLENQISN
LEQSAASQEDLEAMFGNVSYLAHFCRKFQPLYATEAKRVYALEKRMNNY
MQFKSKHRIEPVCLIIRGSPGTGKSLATGIIARAIADKYHSSVYSLPPDPDH
FDGYKQQIVTVMDLCQNPDGKDMSLFCQMVSTVDFIPPMASLEEKGVSF
TSKFVIASTNASNIIVPTVSDSDAIRRRFFMDCDIEVTDSYKTELGRLDAGR
AARLCSENNTANFKRCSPLVCGKAIQLRDRKSKVRYSVDTVVSELIREYN
NRSVIGNTIEALFQ*-COO^-

Figure S2. Enterovirus 2C ATPase assay and 2C constructs used in this study. (A) The standard curve obtained following the protocol of commercial ATPase/GTPase Activity Assay Kit (Sigma-Aldrich, Cat # MAK113). (B) Effects of 250 µM **guanidine**, **dibucaine**, **SFX**, **JX040**, **R523062**, **A967079**, **6aw**, **12a**, **UAWJ172,** and negative control DMSO, on EV-D68 2C (40-329) ATPase activity. (C) Sequences of enterovirus 2C constructs used in this study: CVB3 2C (37-329), EV-D68 2C (40-330), and EV-A71 2C (40-329). The amino acid sequence of the 6xHis tag was colored in red, the tobacco etch virus (TEV) protease cleavage site was colored in blue, and the 2C residues were colored in orange.

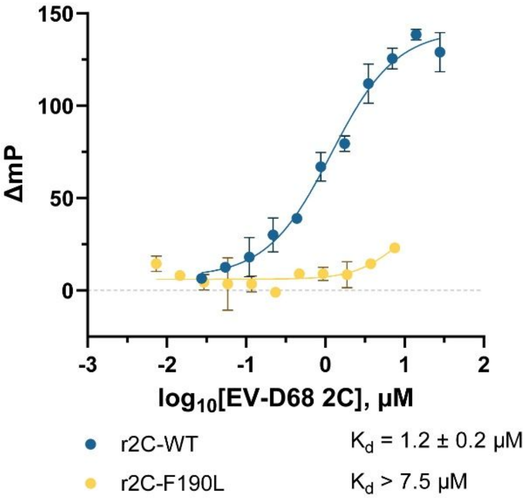

Figure S3. Binding curves of the 50 nM **Jun14157** with increasing concentrations of EV-D68 2C WT and the F190L mutant (r2C-F190L). K_d values are mean ± SD from duplicate experiments.

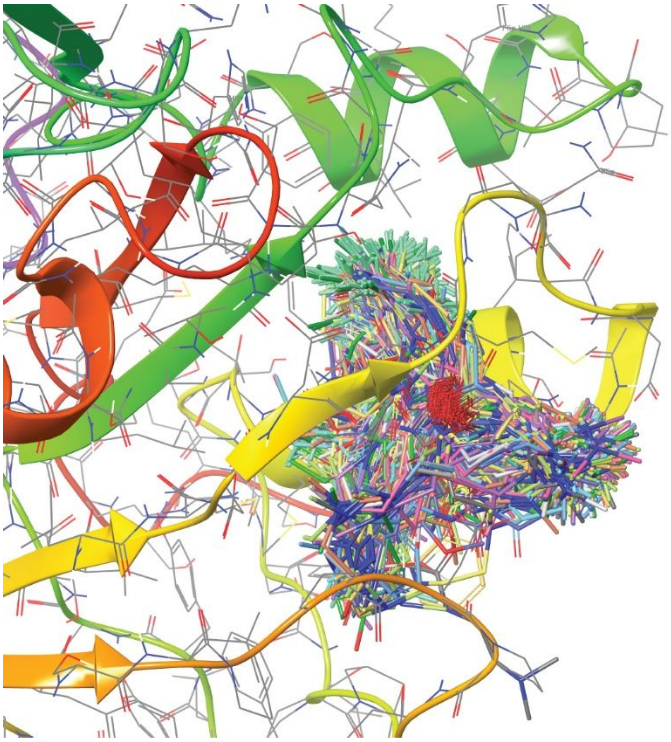

Figure S4. The alignment of top-docking poses from virtual screening with Glide XP.

Table S1. Binding affinities and antiviral activities of reported 2C inhibitors and hits determined by 2C FP assays and CPE assays. For IC_{50} and K_i obtained from FP assays, the plotted values are the mean ± SD (n = 2). For EC_{50} obtained from CPE assays, the plotted values are the mean ± SD (n = 3).

Cpds	IC_{50} (μM) EV-D68 FP	K_i (μM) EV-D68 FP	EC_{50} (nM) EV-D68 CPE	IC_{50} (μM) EV-A71 FP	K_i (μM) EV-A71 FP	EC_{50} (nM) EV-A71 CPE	IC_{50} (μM) CVB3 FP	K_i (μM) CVB3 FP	EC_{50} (nM) CVB3 CPE
**12a**	6.3 ± 0.9	0.7 ± 0.1	74.4 ± 3.5	80.5 ± 6.9	20.8 ± 1.8	4496 ± 247	7.6 ± 0.9	1.3 ± 0.1	110.5 ± 10.0
**Jun571**	11.0 ± 1.0	2.0 ± 0.2	88.5 ± 5.7	9.9 ± 1.8	2.1 ± 0.4	93.6 ± 7.7	14.9 ± 1.6	3.1 ± 0.3	101.2 ± 10.4
**Jun6504**	55.5 ± 5.4	14.2 ± 1.4	340.2 ± 37.0	34.4 ± 2.4	8.6 ± 0.6	987.8 ± 87.7	57.9 ± 6.3	13.6 ± 1.5	3162 ± 140
**6aw**	12.0 ± 2.7	2.2 ± 0.5	99.6 ± 9.4	63.0 ± 7.3	16.2 ± 1.9	2534 ± 401	7.5 ± 0.8	1.3 ± 0.1	265.0 ± 48.6
**JX040**	15.3 ± 4.2	3.1 ± 0.8	78.8 ± 6.9	9.8 ± 1.8	2.1 ± 0.4	731.0 ± 77.0	23.5 ± 3.5	5.2 ± 0.8	250.0 ± 36.4
**A967079**	42.0 ± 4.8	10.5 ± 1.2	371.4 ± 54.6	> 250	> 60	> 5000	76.5 ± 9.8	18.1 ± 2.3	915.1 ± 129.0
**SFX**	152.8 ± 15.0	41.0 ± 4.0	372.7 ± 41.2	> 250	> 60	> 5000	122.0 ± 13.9	29.2 ± 3.3	811.1 ± 155.0
**Telaprevir**	> 250	> 60	943.1 ± 39.1	> 250	> 60	> 5000	> 250	> 60	> 5000
**Pleconaril**	> 250	> 60	53.3 ± 8.3	> 250	> 60	> 5000	> 250	> 60	> 5000
**Dibucaine**	45.8 ± 6.0	11.6 ± 1.5	1832 ± 74	165.0 ± 17.0	43.2 ± 4.5	> 5000	85.13 ± 6.30	20.2 ± 1.5	2800 ± 109
**Guanidine**	> 250	> 60	> 5000	> 250	> 60	> 5000	> 250	> 60	> 5000
**R523062**	> 250	> 60	3990 ± 216	> 250	> 60	> 5000	> 250	> 60	2975 ± 156
**HBB**	> 250	> 60	> 5000	> 250	> 60	> 5000	> 250	> 60	> 5000
**Pirlindole**	> 250	> 60	4415 ± 426	> 250	> 60	> 5000	> 250	> 60	3582 ± 398
**Jun1377**	2.3 ± 0.2	0.5 ± 0.05	9.4 ± 0.8	2.6 ± 0.2	0.2 ± 0.02	89.7 ± 5.8	4.7 ± 0.6	0.6 ± 0.07	29.0 ± 3.3
**Jun15716**	54.5 ± 5.6	15.9 ± 0.6	951.9 ± 66.7	125.0 ± 9.1	44.2 ± 2.6	23661 ± 1230	60.3 ± 7.1	17.8 ± 1.6	719.3 ± 62.7
**Jun15799**	6.3 ± 0.7	0.8 ± 0.1	293.2 ± 23.7	60.7 ± 6.5	21.1 ± 1.7	11454 ± 1005	11.9 ± 1.8	3.0 ± 0.5	288.4 ± 21.6

EV-D68: EV-D68 US/MO/14-18947

EV-A71: EV-A71 Tainan/4643/1998

CVB3: CVB3 Nancy

Spectral Data

HNMR and CNMR spectra of the Intermediate **I-1**

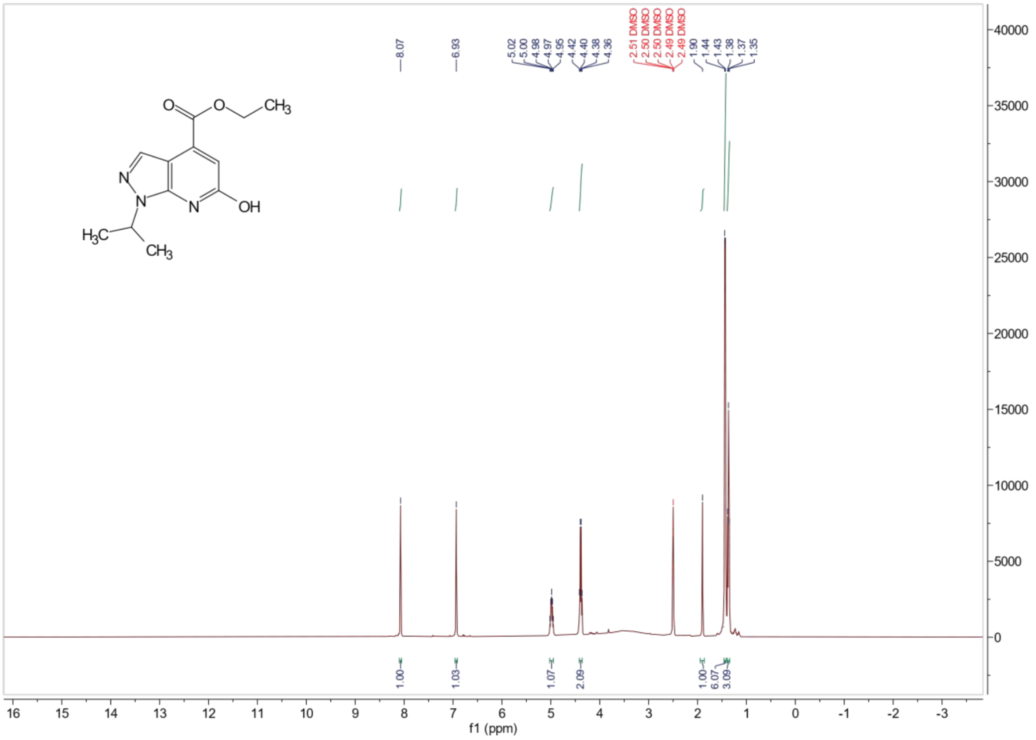

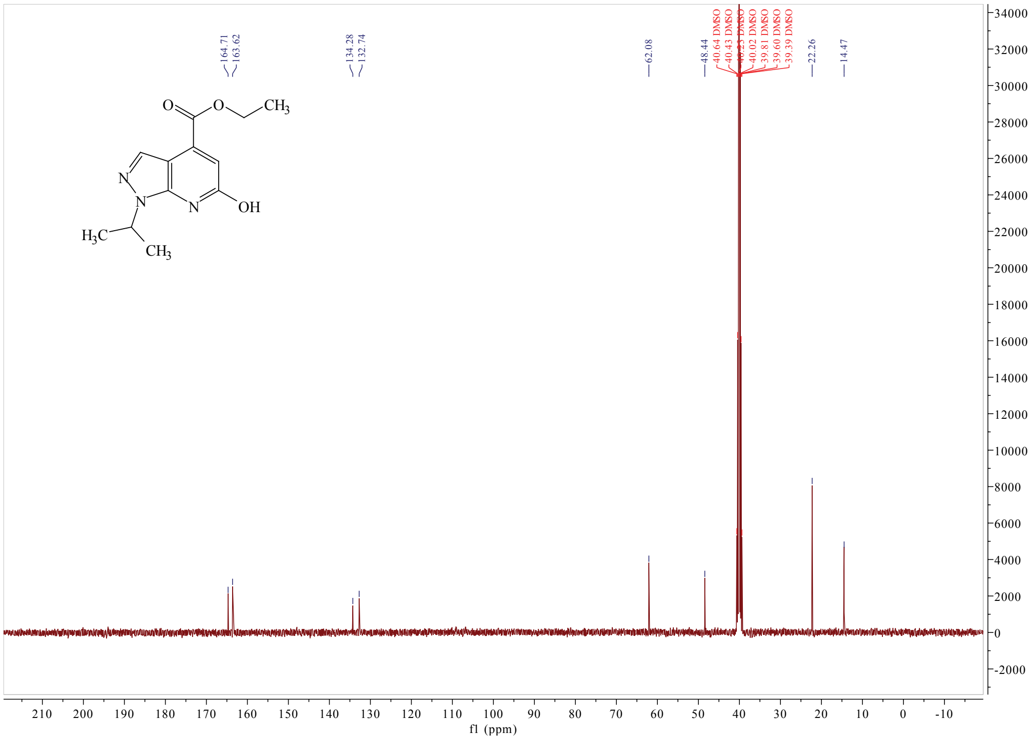

HNMR and CNMR spectra of the Intermediate **I-2**

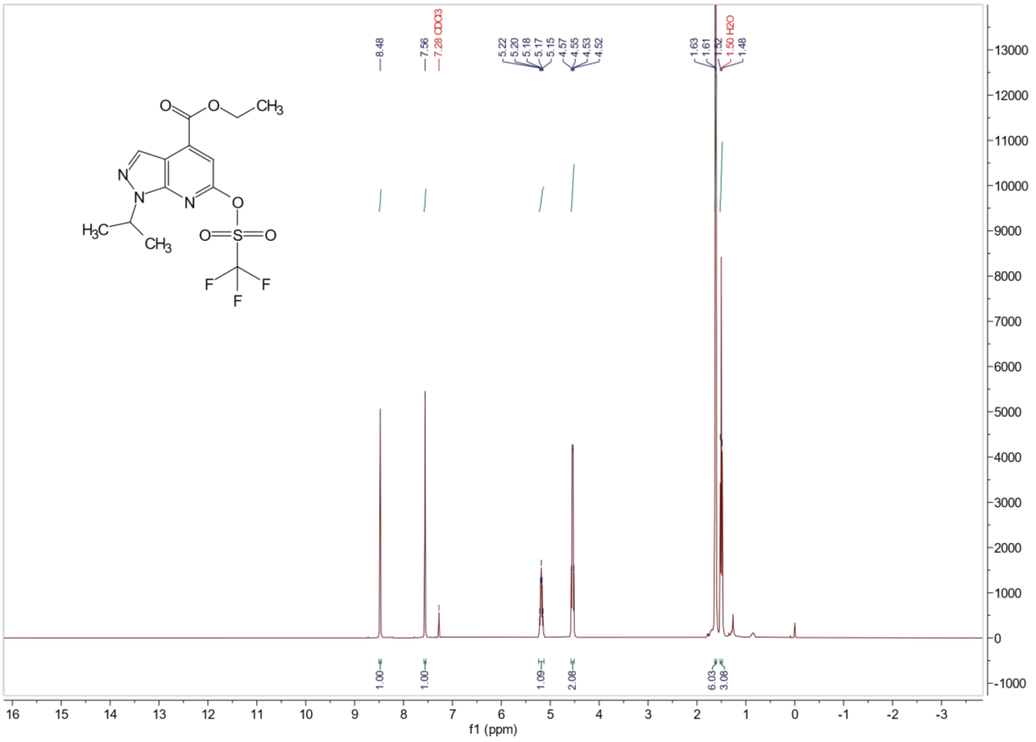

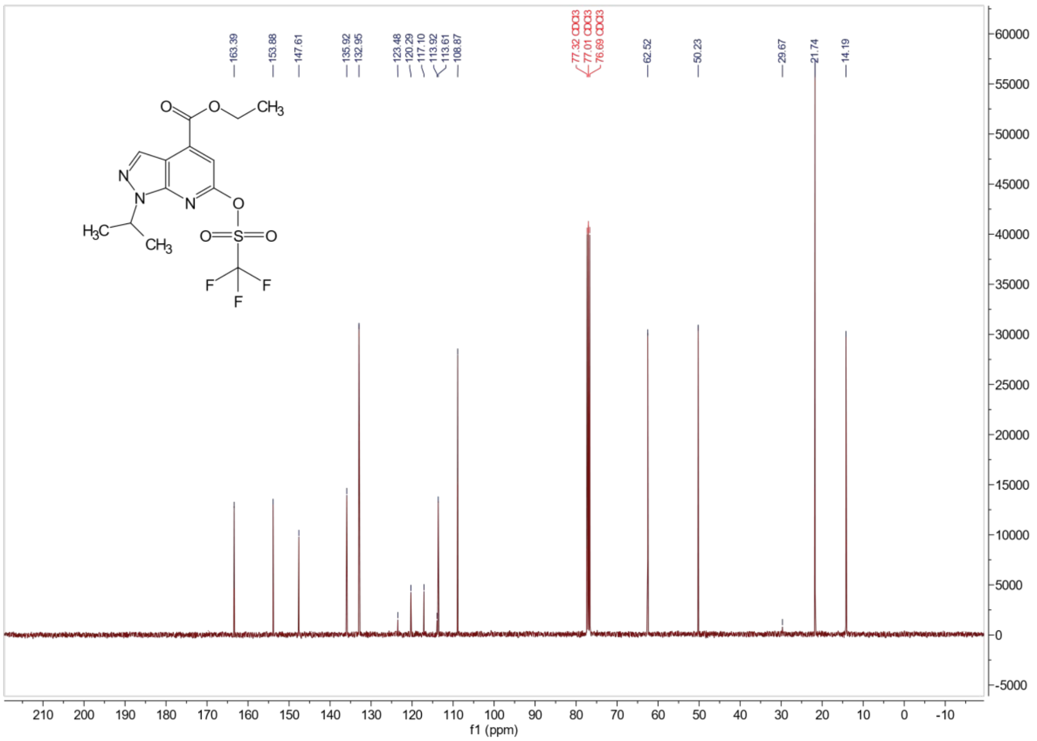

HNMR and CNMR spectra of the Intermediate **I-3**

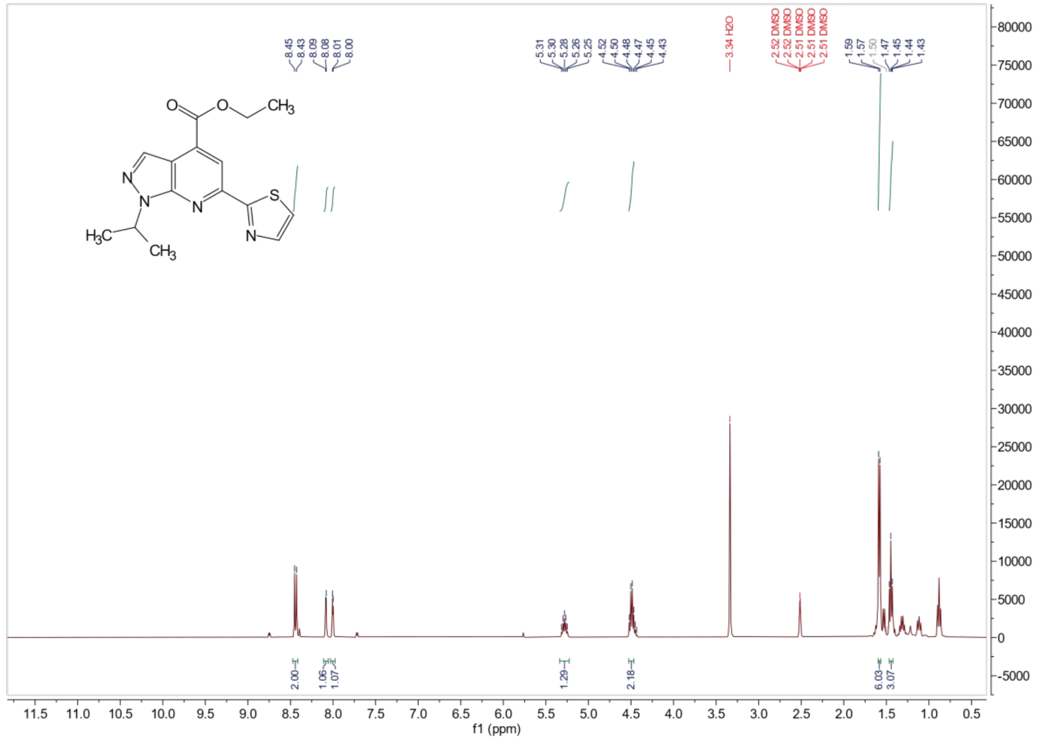

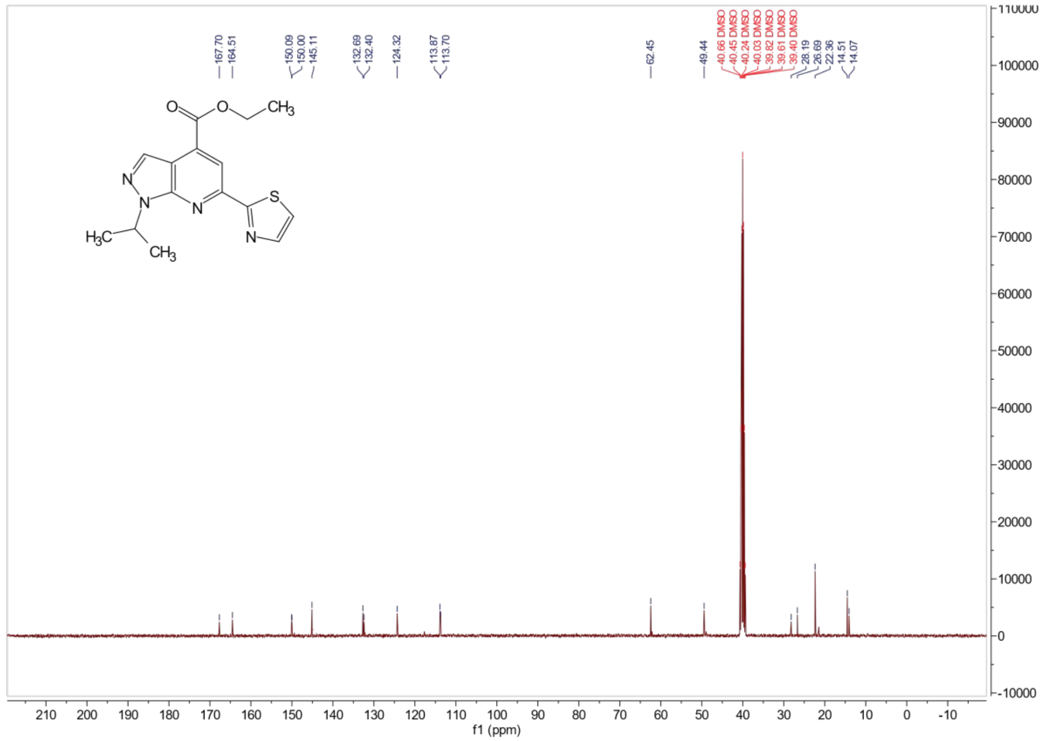

HNMR and CNMR spectra of the Intermediate **I-5**

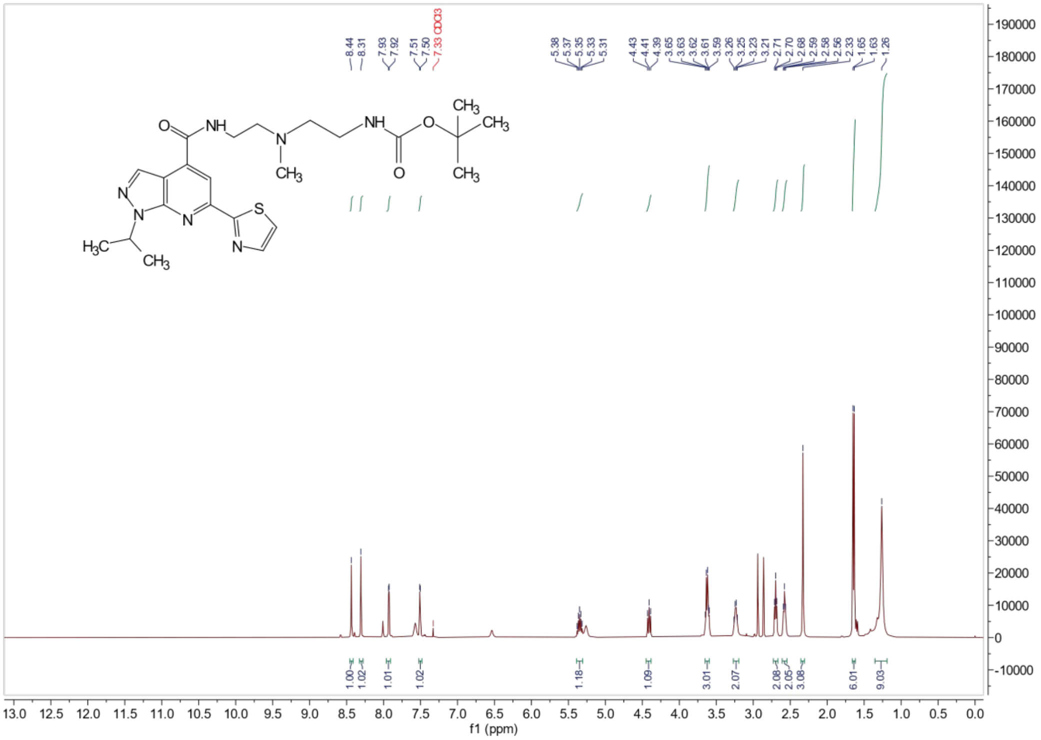

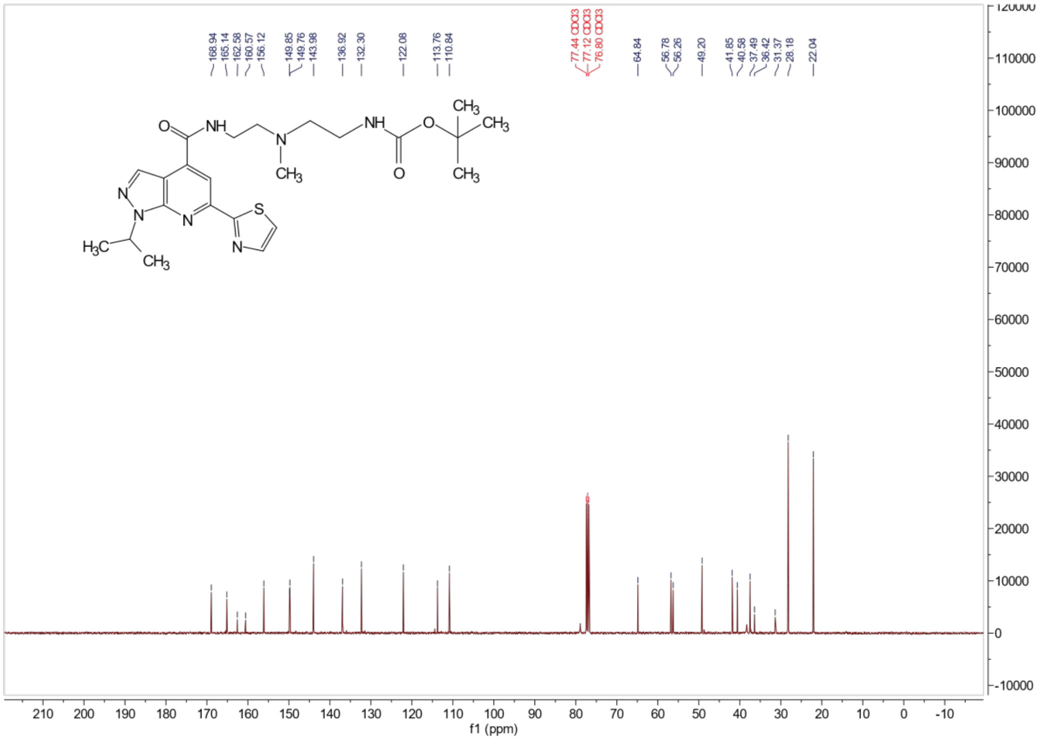

HNMR and CNMR spectra of the Intermediate **Jun14157**

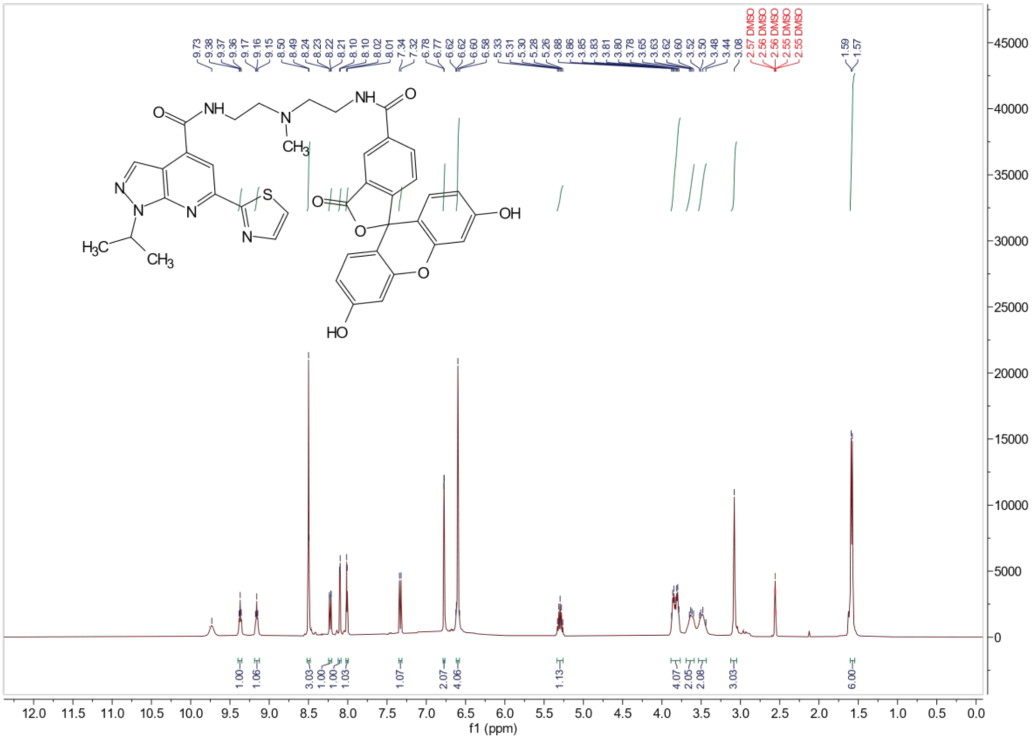

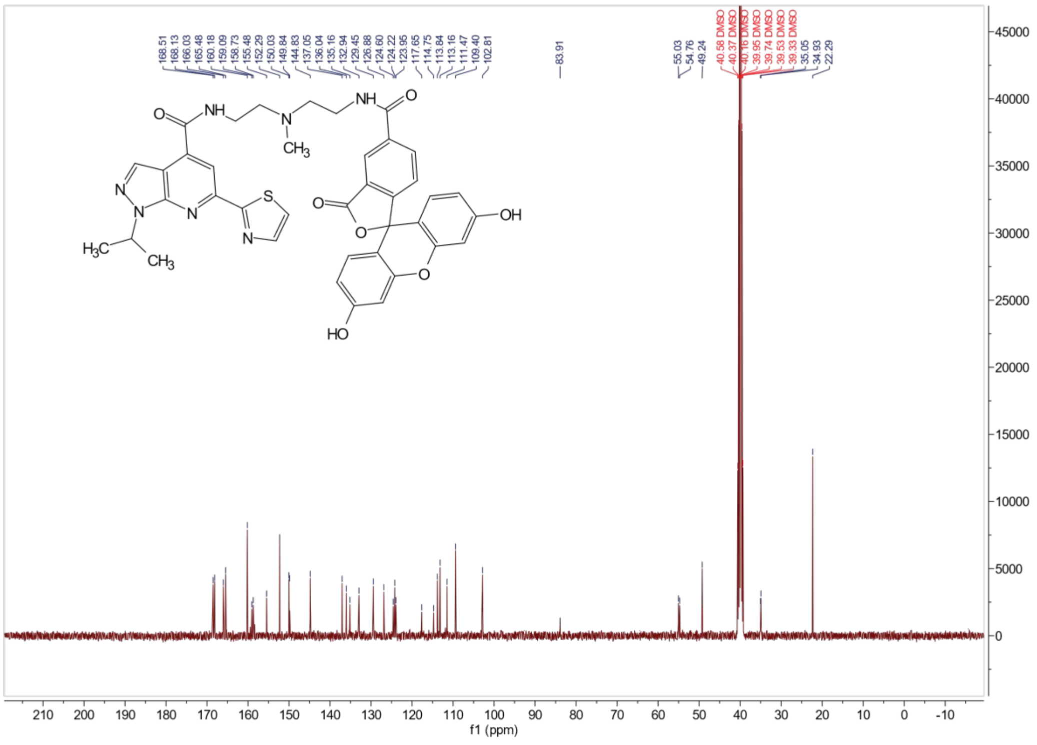

HNMR and CNMR spectra of the Intermediate **Jun1377**

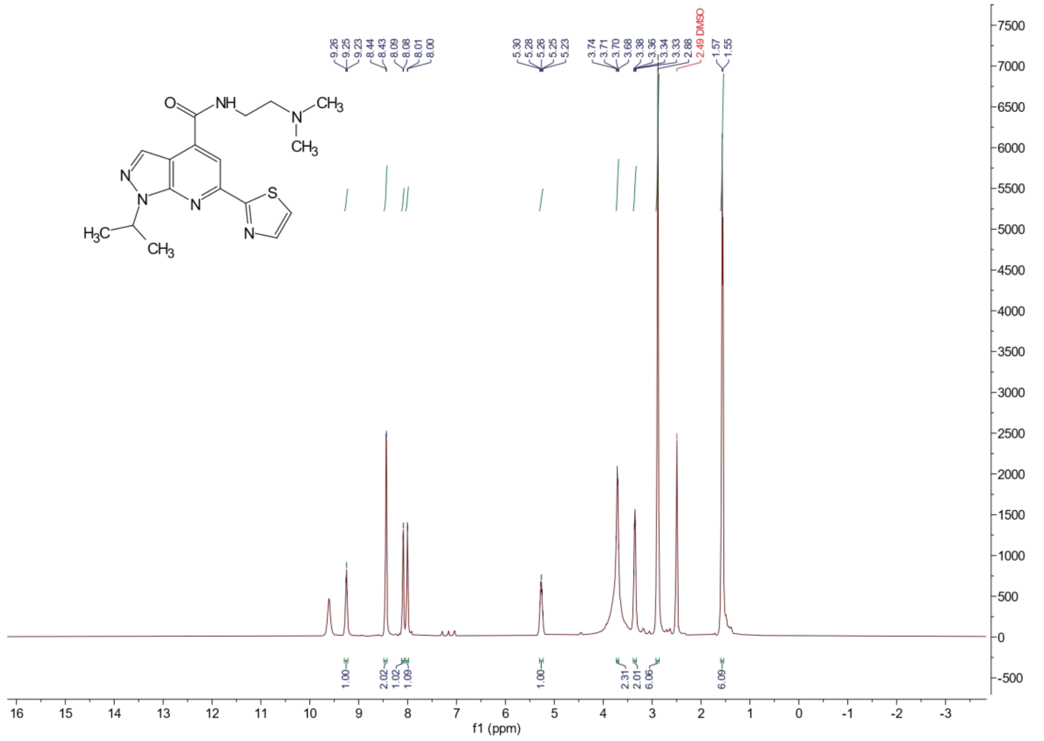

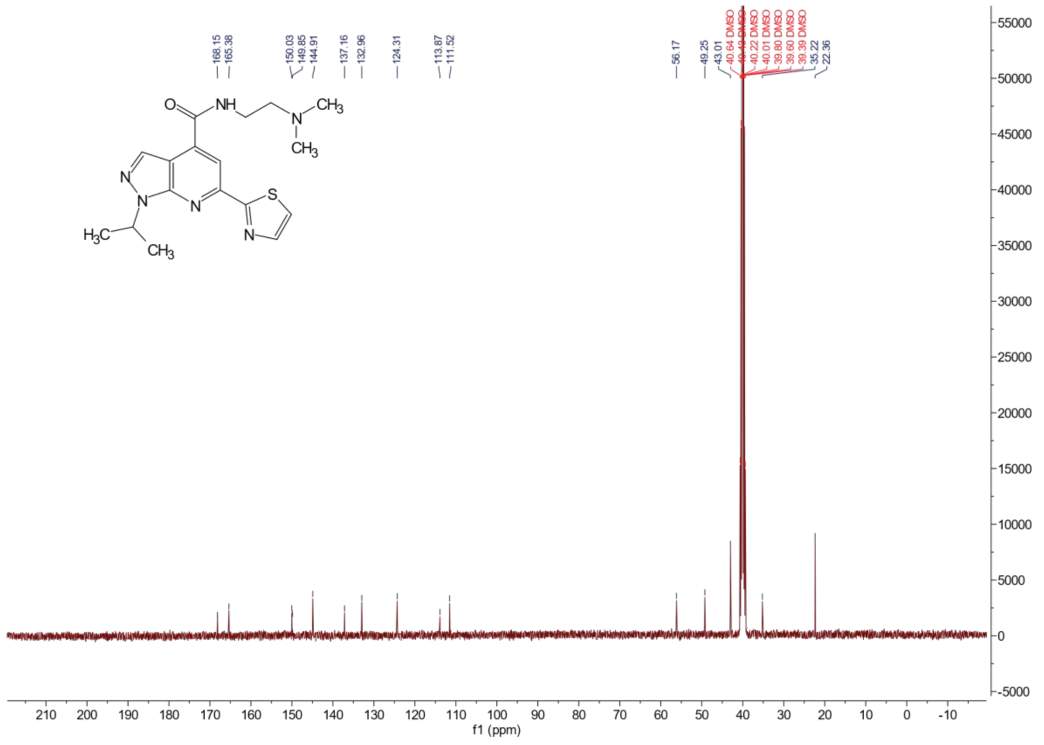

HNMR and CNMR spectra of the Intermediate **Jun15761**

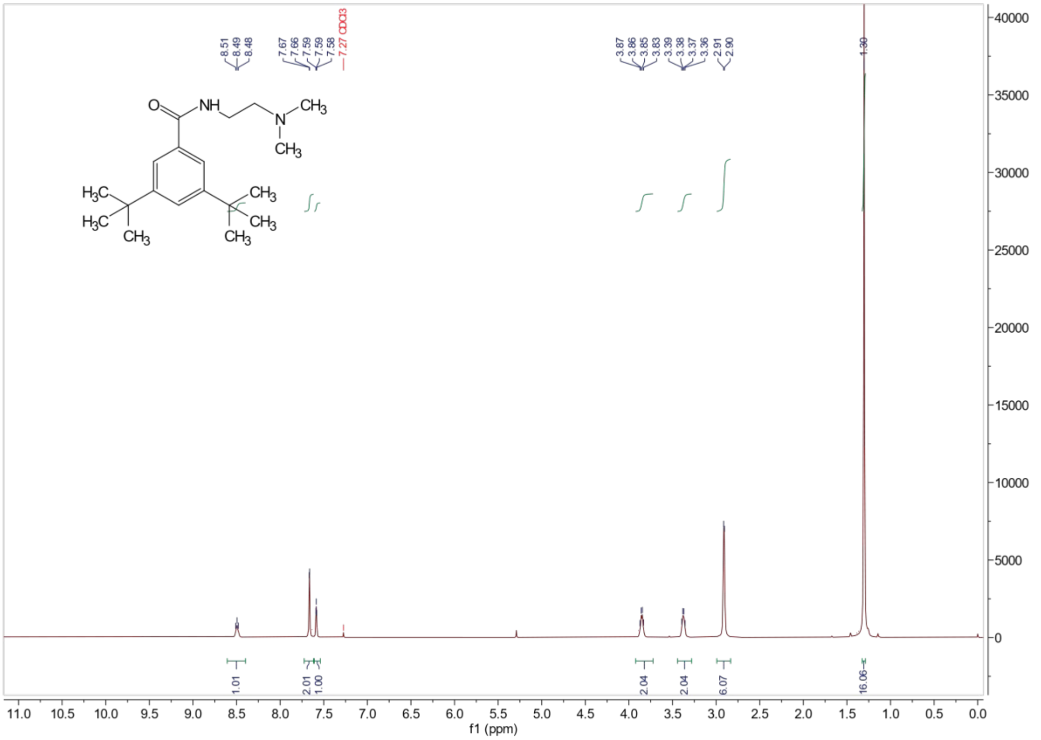

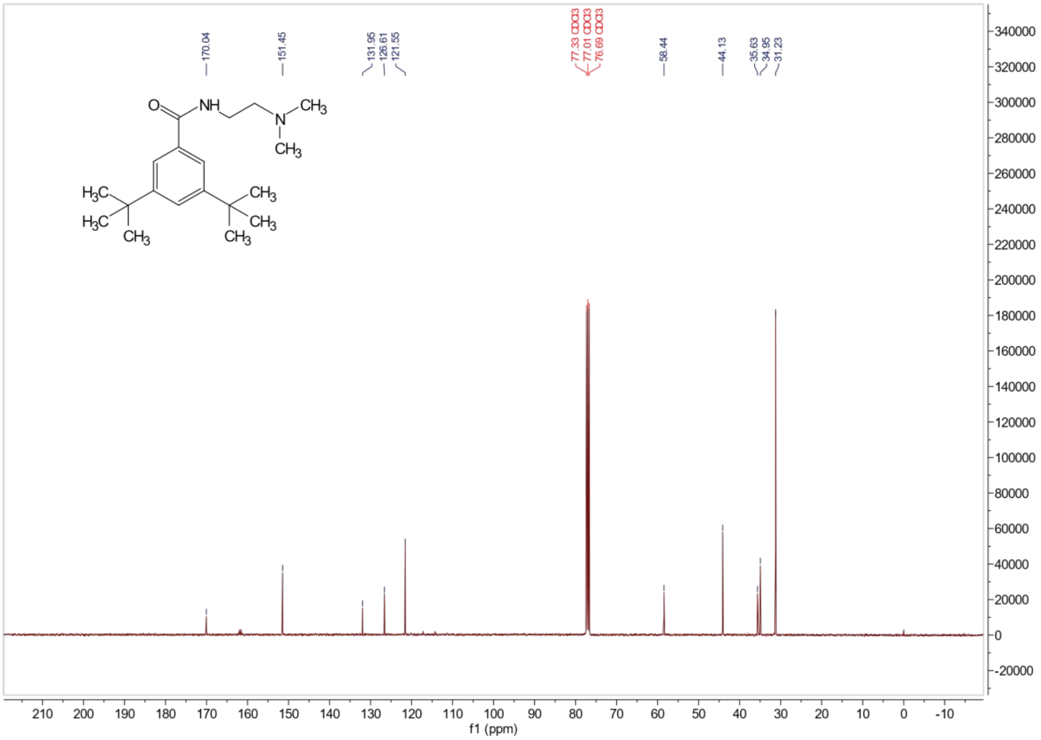

HNMR and CNMR spectra of the Intermediate **Jun15799**

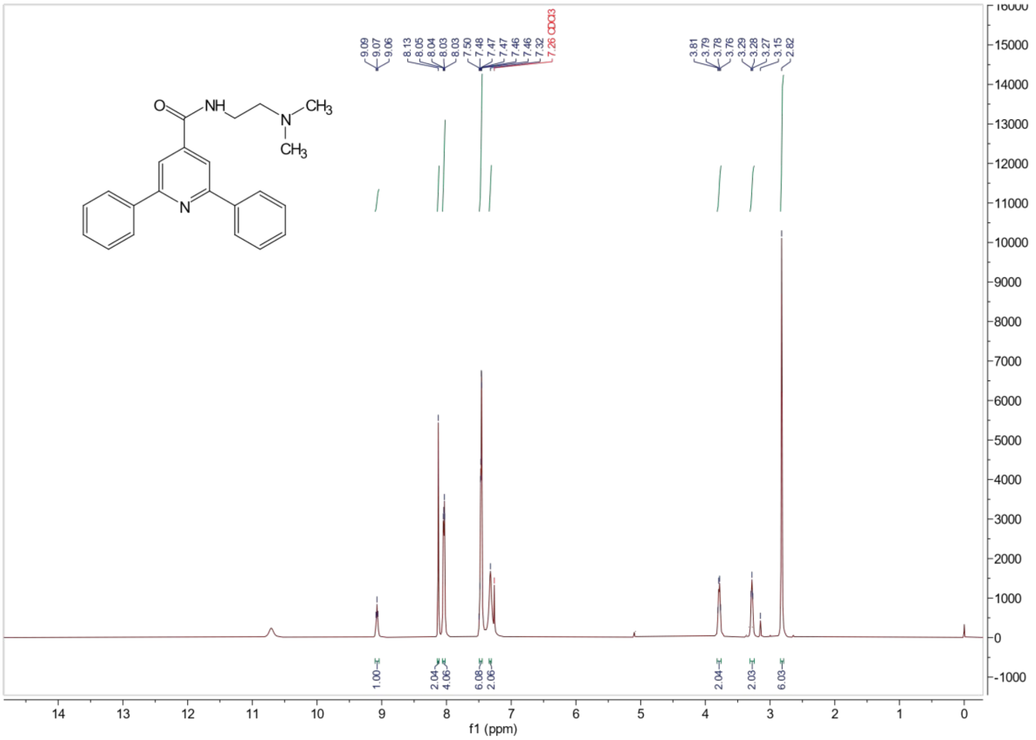

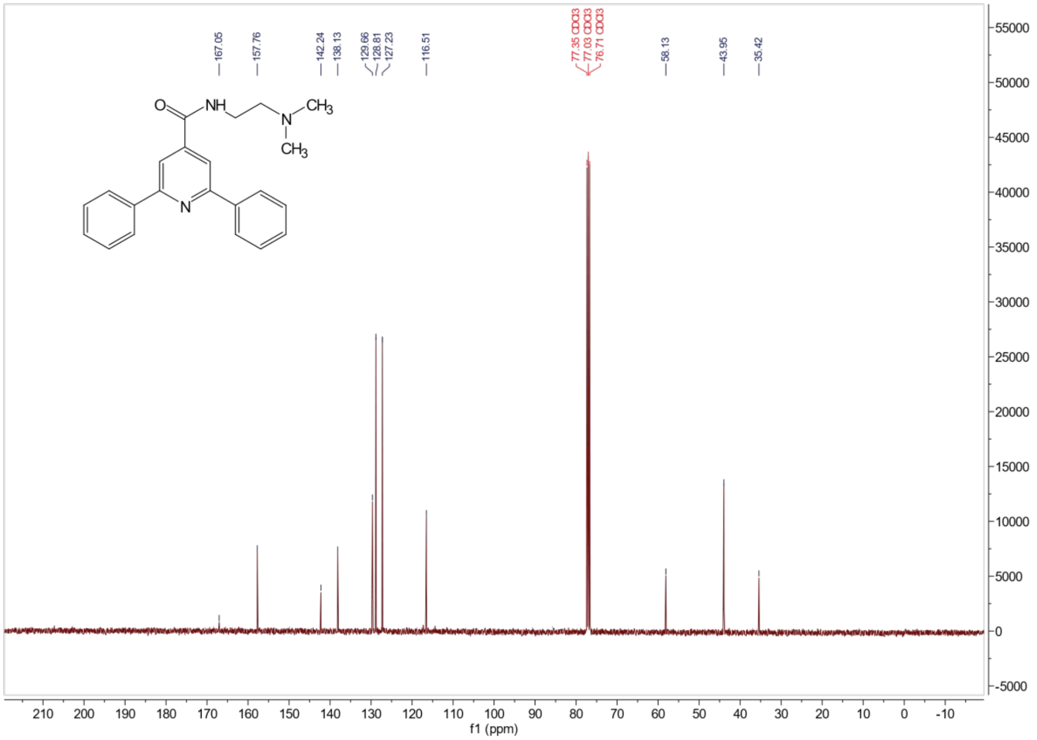

HPLC Trace of Jun1377

Data File C:\CHEM32\1\DATA\BT_SEQN_ _DATE_ _TIME_ 85\041-0201.D
Sample Name: Jun1377

===

Acq. Operator : BT   Seq. Line : 2
Acq. Instrument : Instrument 1   Location : Vial 41
Injection Date : 4/29/2025 2:12:03 AM   Inj : 1
                                        Inj Volume : 5 µl
Sequence File : C:\Chem32\1\DATA\BT_SeqN_ _Date_ _Time_ 85\LSD-RACEMIC SAMPLES-2.S
Method : C:\Chem32\1\DATA\BT_SeqN_ _Date_ _Time_ 85\COLUMNWASHING.M
Last changed : 4/29/2025 2:11:08 AM by BT
(modified after loading)

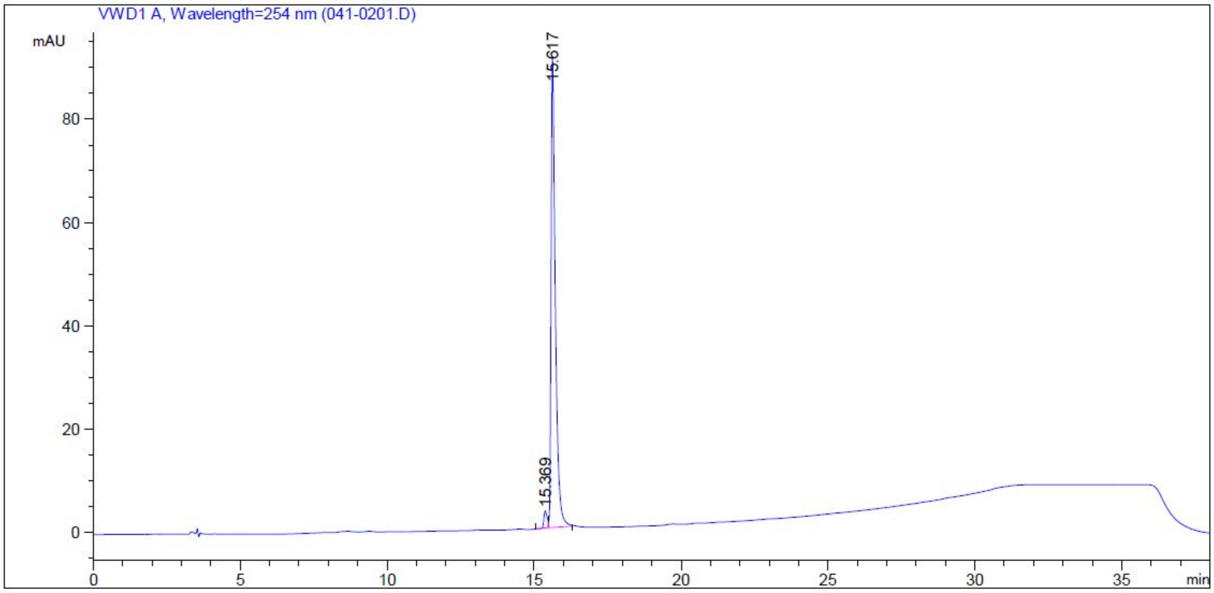

Signal 1: VWD1 A, Wavelength=254 nm

Peak #	RetTime [min]	Type	Width [min]	Area mAU *s	Height [mAU]	Area %
1	15.369	BV	0.1186	26.60329	3.36036	2.8023
2	15.617	VB	0.1457	922.72644	91.23964	97.1977

HPLC Trace of Jun14157

Data File C:\CHEM32\1\DATA\BT_SEQN_ _DATE_ _TIME_ 85\042-0301.D
Sample Name: Jun14157

===

Acq. Operator : BT Seq. Line : 3
Acq. Instrument : Instrument 1 Location : Vial 42
Injection Date : 4/29/2025 2:51:11 AM Inj : 1
Inj Volume : 5 µl
Sequence File : C:\Chem32\1\DATA\BT_SeqN_ _Date_ _Time_ 85\LSD-RACEMIC SAMPLES-2.S
Method : C:\Chem32\1\DATA\BT_SeqN_ _Date_ _Time_ 85\COLUMNWASHING.M
Last changed : 4/29/2025 2:50:21 AM by BT
(modified after loading)

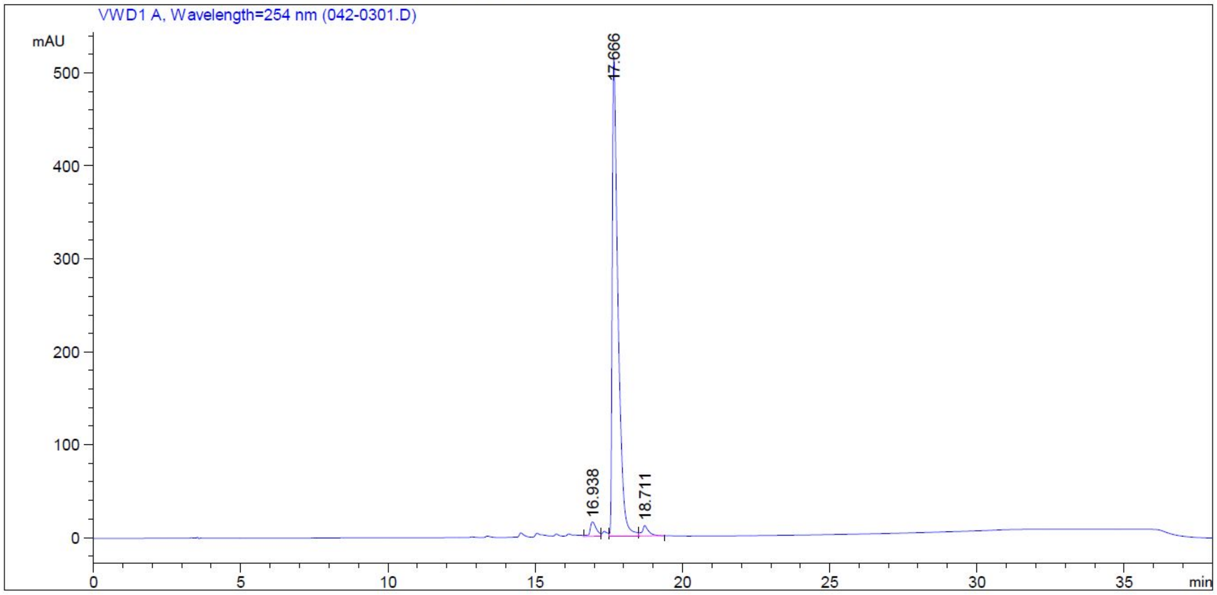

Signal 1: VWD1 A, Wavelength=254 nm

Peak #	RetTime [min]	Type	Width [min]	Area mAU *s	Height [mAU]	Area %
1	16.938	VV	0.2248	140.61835	15.75439	1.9396
2	17.666	VV	0.1955	7030.32861	515.50269	96.9448
3	18.711	VB	0.2128	80.87888	11.39643	1.1156

HPLC Trace of Jun15716

```
Data File C:\CHEM32\1\DATA\BT\_SEQN_ _DATE_ _TIME_ 85\043-0401.D
Sample Name: Jun15716

    =====================================================================
    Acq. Operator   : BT                              Seq. Line :   4
    Acq. Instrument : Instrument 1                     Location : Vial 43
    Injection Date  : 4/29/2025 3:30:18 AM                  Inj :   1
                                                   Inj Volume : 5 µl
    Acq. Method     : C:\Chem32\1\DATA\BT\_SeqN_ _Date_ _Time_ 85\COLUMNWASHING.M
    Last changed    : 4/29/2025 3:29:28 AM by BT
                      (modified after loading)
    Analysis Method : C:\CHEM32\1\DATA\BT\_SEQN_ _DATE_ _TIME_ 85\043-0401.D\DA.M (COLUMNWASHING.M)
    Last changed    : 4/29/2025 5:13:44 AM by BT
```

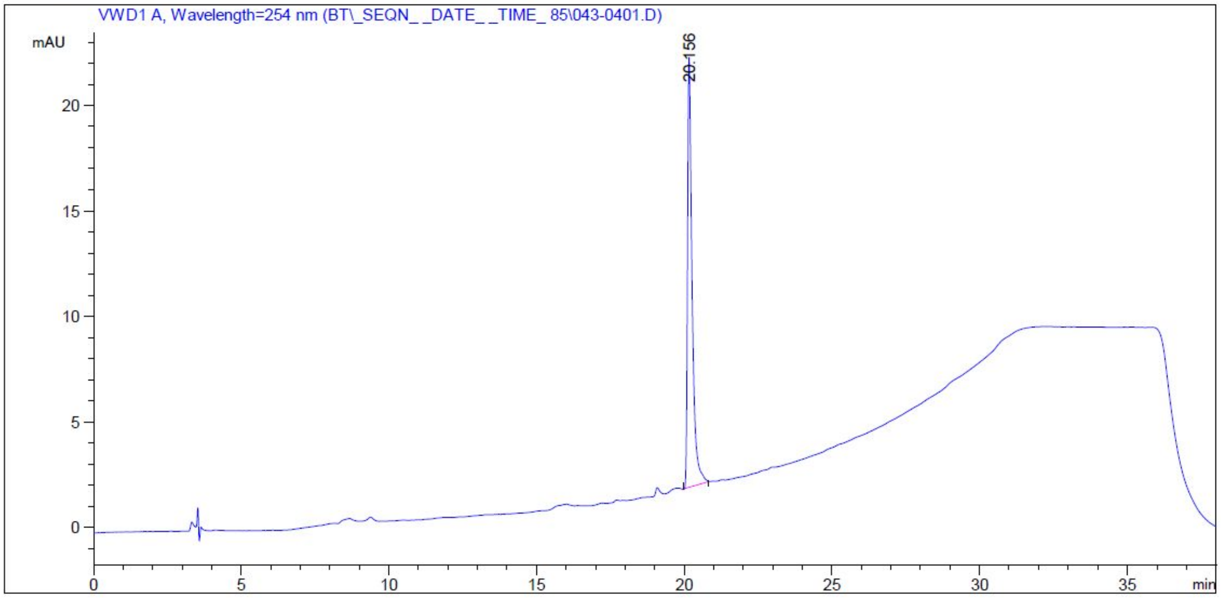

Peak #	RetTime [min]	Type	Width [min]	Area mAU *s	Height [mAU]	Area %
1	20.156	BB	0.1550	220.17819	20.40146	100.0000

HPLC Trace of Jun15799

Data File C:\CHEM32\1\DATA\BT_SEQN_ _DATE_ _TIME_ 85\044-0501.D
Sample Name: Jun15799

```
=====================================================================
Acq. Operator   : BT                              Seq. Line :    5
Acq. Instrument : Instrument 1                     Location : Vial 44
Injection Date  : 4/29/2025 4:09:29 AM                  Inj :    1
                                                 Inj Volume : 5 µl
Sequence File   : C:\Chem32\1\DATA\BT\_SeqN_ _Date_ _Time_ 85\LSD-RACEMIC SAMPLES-2.S
Method          : C:\Chem32\1\DATA\BT\_SeqN_ _Date_ _Time_ 85\COLUMNWASHING.M
Last changed    : 4/29/2025 4:08:34 AM by BT
                  (modified after loading)
```

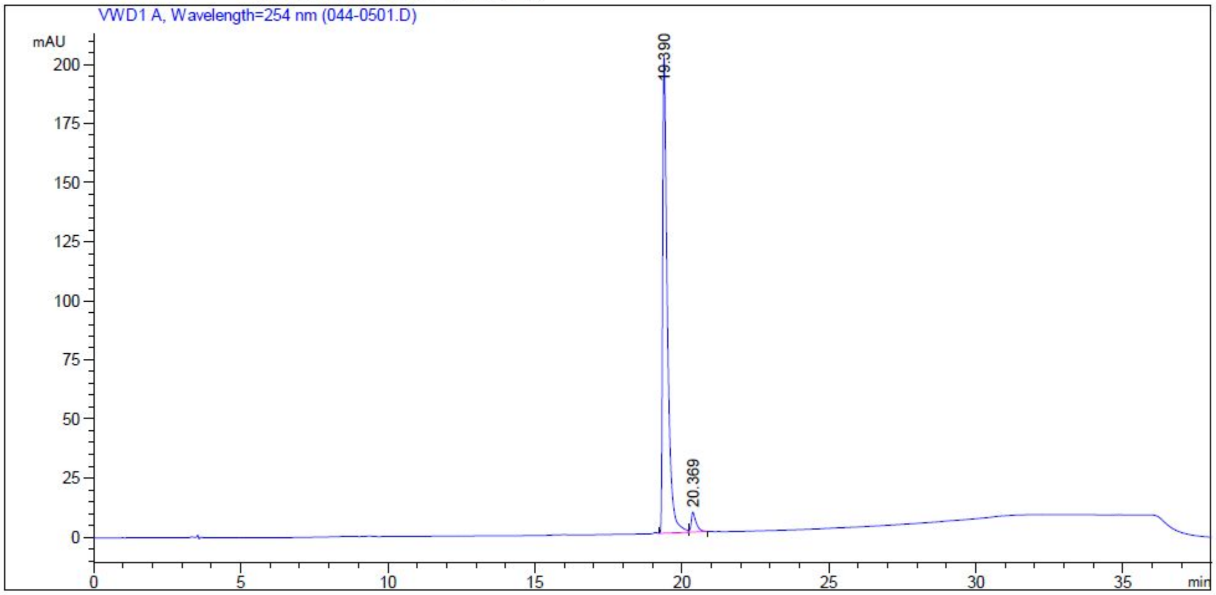

Peak #	RetTime [min]	Type	Width [min]	Area mAU *s	Height [mAU]	Area %
1	19.390	VV	0.1627	2279.72778	201.30348	95.7680
2	20.369	VB	0.1671	100.74022	8.52056	4.2320
